# SAMD9L inhibits flavivirus translation independently of its capacity to trigger innate immune response

**DOI:** 10.1371/journal.ppat.1013773

**Published:** 2025-12-08

**Authors:** Marion Cannac, Jim Zoladek, Inès Bribes, Mathis Fresneau--Resende, Alexandre Legrand, Rémi Demeure, Eva Zusinaite, Andres Merits, Lucie Etienne, Sébastien Nisole

**Affiliations:** 1 Institut de Recherche en Infectiologie de Montpellier (IRIM), Univ Montpellier, CNRS UMR9004, INSERM, Montpellier, France; 2 Pathogenesis and Control of Chronic and Emerging Infections (PCCEI), INSERM, Etablissement Français du Sang, Univ Montpellier, Montpellier, France; 3 Centre international de recherche en infectiologie (CIRI), Inserm U1111, Université Claude-Bernard Lyon 1, CNRS UMR5308, École normale supérieure de Lyon, Lyon, France; 4 Institute of Bioengineering, University of Tartu, Tartu, Estonia; Universite de Reims Champagne-Ardenne UFR de Medecine, FRANCE

## Abstract

Interferon-stimulated genes (ISGs) play a pivotal role in the innate immune response to viral infection. Among them, SAMD9 and its paralog SAMD9L have recently emerged as important antiviral effectors with translation-inhibitory activity. While both proteins restrict poxvirus, rotavirus and reovirus replication, only SAMD9L has been shown to inhibit HIV and other lentiviruses. In this study, we identify human SAMD9L as a potent and broad-spectrum restriction factor that targets multiple medically relevant flaviviruses, including West Nile virus (WNV), Zika virus (ZIKV), dengue virus (DENV), and Usutu virus (USUV). Exogenous expression of SAMD9L, but not SAMD9, efficiently suppressed replication of all tested flaviviruses. Furthermore, its knockdown in human myeloid cells, including microglial cells and primary macrophages, impaired the antiviral activity of type I interferon, identifying SAMD9L as a key antiviral ISG in primary target cells of flavivirus infection. Mechanistically, we demonstrate that SAMD9L inhibits viral replication by targeting the translation of flaviviral RNA, and that this activity depends on its Schlafen-like ribonuclease domain, previously implicated in the inhibition of HIV-1 translation. Interestingly, although SAMD9 does not inhibit flavivirus replication, it is able to repress the translation of flaviviral RNA outside the context of infection, suggesting that its activation may be virus-specific or that flaviviruses have evolved mechanisms to evade or counteract SAMD9’s antiviral activity. Finally, we confirm that SAMD9 and SAMD9L overexpression induces activation of the innate immune response. However, this immunostimulatory function is dispensable for SAMD9L-mediated antiviral activity, since SAMD9L is able to restrict flavivirus replication independently of innate immune activation. Together, our findings broaden the known antiviral repertoire of SAMD9L, establish its essential role in restricting flavivirus replication via translational repression, and highlight its function as a key component of the cellular defenses against flaviviruses in myeloid cells.

## Author summary

Flaviviruses are a group of viruses that include West Nile, Zika, dengue, Usutu, and tick-borne encephalitis viruses. They are transmitted by arthropods, either mosquitoes or ticks, and can cause serious and sometimes fatal illnesses as well as long-term complications. The body’s first line of defense against these infections is the innate immune system, which quickly expresses protective proteins in response to invading viruses. In this study, we investigated one of these proteins, called SAMD9L, and found that it plays a key role in protecting human cells from flavivirus infection. Human SAMD9L stops the virus from making its own proteins, which is a necessary step for the virus to multiply. This antiviral effect was especially strong in innate immune cells that are natural targets of flaviviruses. We also found that although human SAMD9L can trigger immune responses, its ability to block flavivirus replication does not rely on this function. These findings reveal a direct way in which human SAMD9L helps control flavivirus infections and highlight its importance in the body’s natural defense against viruses.

## Introduction

The interferon (IFN) response is a pivotal component of the innate immune system, playing a critical role in the defense mechanisms against viral infections. Upon detection of viral pathogens by pattern recognition receptors (PRRs), infected cells secrete type I interferons (IFN-I), primarily IFN-α and IFN-β. These cytokines initiate a broad antiviral program by binding to IFN receptors on both infected and surrounding cells, triggering a signaling cascade that activates the expression of interferon-stimulated genes (ISGs) [1,2]. ISGs can inhibit multiple steps of the viral replication cycle, including viral entry into host cells, replication of viral genetic material, and assembly and budding of newly formed virions. Viral translation, a process entirely dependent on the host cell machinery, represents a critical vulnerability in viral replication that is specifically targeted by several ISGs, including *eukaryotic translation initiation factor 2 alpha kinase 2* (*EIF2AK2*, better known as *PKR*) [3], *zinc finger CCCH-type containing, antiviral 1* (*ZC3HAV1*, better known as *ZAP*) [4], *interferon-induced proteins with tetratricopeptide repeats* (*IFIT*s) [5], and *Schlafen 11* (*SLFN11*) [6]. While most ISGs block viral translation by targeting key stages in the translation process, such as disrupting initiation or binding viral RNA to prevent its translation, SLFN11 employs an alternative mechanism [2,7]. Instead of directly interfering with the translation machinery, SLFN11 degrades specific tRNAs, thereby inhibiting viral protein synthesis [6]. Interestingly, another ISG named Sterile alpha motif domain-containing proteins 9 (SAMD9) was recently found to display a similar mechanism of action, interfering with the translation of poxviruses by limiting the available pool of tRNA [8]. However, while SLFN11 only degrades rare tRNAs, specifically disrupting the translation of proteins with atypical codon usage such as HIV-1 proteins [6], human SAMD9 was reported to cleave the essential tRNA^Phe^, thereby causing a global translational arrest [8]. Interestingly, its anticodon nuclease activity was found to be triggered by poxvirus infection, suggesting that human SAMD9 could function as both a viral sensor and an antiviral effector [8].

SAMD9 has a paralog named SAMD9-like (SAMD9L), encoded by an adjacent gene that likely arose from a duplication event early in mammalian evolution [9,10]. Some species have retained both genes, while others have lost one of them. For example, mice lack SAMD9, whereas other mammals lack SAMD9L [9,10]. The absence of one paralog in certain species suggests that SAMD9 and SAMD9L may fulfill overlapping functions. Human SAMD9 and SAMD9L share approximately 60% amino-acid sequence identity and exhibit the same domain architecture [9,11,12]. They are involved in several essential cellular processes, including cell proliferation [13], inflammation [14], stress response [13,15], apoptosis [13], endosomal trafficking [16,17] and protein translation [13,18,19]. Beyond their critical role in normal cellular functions, SAMD9 and SAMD9L are also involved in several severe genetic disorders, including MIRAGE syndrome, ataxia-pancytopenia (ATXPC) syndrome, myeloid leukemia syndrome with monosomy 7 (MLSM7), refractory cytopenia of childhood (RCC), or SAMD9L-associated autoinflammatory disease (SAAD) [13,18,20–24]. Most of these genetic disorders arise from germline gain-of-function mutations in SAMD9 or SAMD9L, which lead to excessive translational repression, thus disrupting normal cellular growth and functions. Translational inhibition is also central to the antiviral functions of SAMD9 and SAMD9L. Human SAMD9 and SAMD9L, as well as murine SAMD9L, restrict poxvirus replication [8,15,25,26]. In addition, human SAMD9 and murine SAMD9L inhibit reoviruses and rotaviruses [27]. Human SAMD9L has also been shown to inhibit the replication of HIV-1 and other lentiviruses, in a lentiviral-strain specific manner [28]. However, this antiviral effect does not extend to all viruses, as exogenously expressed human SAMD9L fails to restrict murine leukemia virus (MLV) or *Mopeia mammarenavirus* (MOPV) [28]. These findings either challenge the notion that SAMD9 and SAMD9L induce a broad, non-specific shutdown of translation [8], or suggest that certain viruses have evolved specific strategies to evade or counteract their antiviral activity. Whether other viruses are susceptible to inhibition by SAMD9 and/or SAMD9L is not known.

In a recent study aimed at discovering new ISGs that disrupt the replication of West Nile virus (WNV) and Usutu virus (USUV), two closely related mosquito-borne neurotropic flaviviruses, we conducted an expression screen of several hundred human ISGs and identified SAMD9L as one of the top hits [29]. Flaviviruses are a group of positive-sense, single-stranded RNA viruses within the *Flaviviridae* family, primarily transmitted by hematophagous arthropods, such as mosquitoes and ticks. Among the many flaviviruses identified, several pose significant threats to human health, including dengue virus (DENV), yellow fever virus (YFV), Zika virus (ZIKV) and WNV, which are responsible for a range of serious and often debilitating diseases [30]. Other flaviviruses are emerging in various parts of the world, including Japanese encephalitis virus (JEV), USUV, and tick-borne encephalitis virus (TBEV), posing a growing challenge to public health.

Several ISGs play a crucial role in inhibiting flavivirus replication. Among the most effective are IFITMs, which block viral entry [31–34], ISG20, which degrades viral RNA [31,35,36], and IFI6, which disrupts the formation of viral replication factories [32,37,38]. The translation step of flavivirus life cycle is also targeted by various ISGs, including IFITs [39–41], SHFL [42,43], and SLFN11 [44]. However, although SAMD9L was identified as a hit in a screen of ISGs inhibiting WNV replication [45], its anti-flavivirus activity has not yet been formally investigated.

Here, we demonstrate that human SAMD9L, but not its paralog SAMD9, acts as a broad-spectrum restriction factor that inhibits the replication of flaviviruses, including DENV, ZIKV, WNV, and USUV. Using WNV as a model, we show that endogenous SAMD9L plays a critical role in mediating the antiviral effects of IFN-I in myeloid cells, including microglial cells and primary macrophages. As previously described for poxviruses [15,25,26] and HIV-1 [28], we identify viral translation as the key step of the flavivirus life cycle targeted by SAMD9L. Furthermore, we demonstrate that this restriction mechanism relies on the Schlafen-like ribonuclease domain of SAMD9L, as observed for HIV-1 and poxviruses. Finally, although SAMD9L overexpression can activate innate immune response, as recently reported [27], restriction of flavivirus translation is independent of this activity. Together, our findings reveal that SAMD9L’s antiviral spectrum extends to flaviviruses, establishing it as a potent ISG against viruses of public health importance.

## Results

### Human SAMD9L, but not its paralog SAMD9, restricts flavivirus replication

**Fig 1:**
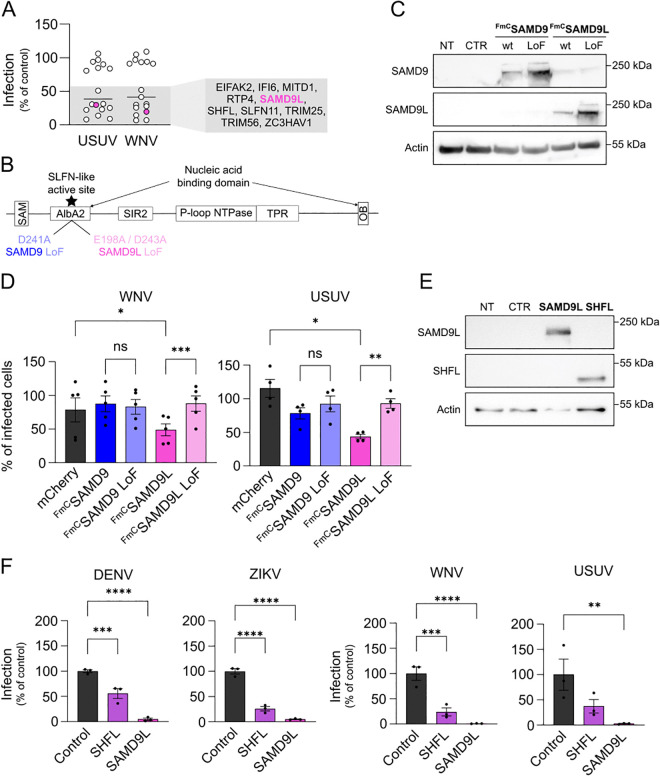
SAMD9L restricts replication of multiple mosquito-borne flaviviruses. **(A)** HEK293T cells were transduced with individual lentiviral vectors encoding different ISGs for 72 h, followed by infection with WNV or USUV at a MOI of 1 for 48 h. Infection and transduction efficiencies were assessed by flow cytometry. Infection rates are shown as percentages normalized to the empty vector control. Each dot represents the mean of two biological replicates per ISG. **(B)** Schematic representation of SAMD9 and SAMD9L protein domain architecture. Point mutations used to generate the catalytically inactive (LoF) variants are indicated. **(C)** Validation of overexpression of N-terminally FLAG-mCherry-tagged (FmC) SAMD9, SAMD9 LoF, SAMD9L, and SAMD9L LoF in HEK293T cells by Western blot, 48 h post-transfection. **(D)** HEK293T cells were transfected with the indicated plasmids for 48 h and subsequently infected with WNV at an MOI of 1 for 48 h. Infection and transfection efficiencies were assessed by flow cytometry. Infection levels are shown in transfected (mCherry^+^) cells relative to the non-transfected (mCherry^-^) cells of the same well (internal control). **(E)** Validation of SAMD9L and SHFL expression in Vero E6 cells 72 h post-transduction with lentiviral vectors by Western blot. **(F)** Vero E6 cells were transduced with lentiviruses encoding untagged SHFL or SAMD9L for 72 h, then infected with DENV-2 or ZIKV for 72 h, or with WNV or USUV for 48 h (all at a MOI of 1). Infection and transduction efficiencies were measured by flow cytometry, and infection levels were normalized to the empty control vector. Each dot represents a biological replicate. Data are presented as mean ± SEM. Statistical analyses: one-way ANOVA with Fisher’s multiple comparisons test (**D**) or with Dunnett’s multiple comparisons test **(F)**. Statistical significance: ****, p ≤ 0.0001; ***, p ≤ 0.001; **, p ≤ 0.01; *, p ≤ 0.05; ns, p > 0.05. Abbreviations: CTR, control; NT, non-transduced/non-transfected.

In order to identify antiviral effectors that inhibit USUV and WNV replication, we recently screened a library of lentiviral vectors encoding 570 human ISGs in HEK293T cells, and pinpointed SAMD9L as one of the best candidates [29]. To confirm its antiviral activity against these two flaviviruses, we carried out a secondary screen with 10 of the most promising hits, as well as 7 control ISGs that showed no activity (Fig 1A). Again, SAMD9L emerged as one of the most potent antiviral ISGs, alongside well-known anti-flavivirus factors such as SHFL, IFI6, MITD1, RTP4, and SLFN11 [29,32,37,42,44,46] (Fig 1A).

Given that human SAMD9L shares with its paralog SAMD9 the ability to inhibit translation through ribonuclease activity, and that both proteins suppress poxvirus replication [8,15,25,26], we investigated whether human SAMD9 also inhibits flavivirus replication. Alongside wild-type (wt) SAMD9L and SAMD9, we included mutants of these proteins carrying loss-of-function (LoF) mutations in their Schlafen-like ribonuclease domain, which were previously shown to abolish both their enzymatic and antiviral activities [28] (Fig 1B). Thus, HEK293T cells were transfected with plasmids encoding wt or LoF mutant SAMD9/SAMD9L proteins, all fused at the N-terminus to a FLAG-mCherry (FmC) tag to facilitate detection. Western blot analysis confirmed expression of both wt and LoF proteins, with the LoF mutant consistently detected at higher levels than the wt protein under the conditions tested (Fig 1C). Following transfection, cells were infected with WNV (lineage 2) or USUV (Africa 2 lineage), and infection rates were assessed 48 h post-infection by flow cytometry using a pan-flavivirus anti-envelope antibody. As expected, wt SAMD9L inhibited both WNV and USUV replication, whereas the LoF mutant protein had no effect (Fig 1D). In contrast, neither wt nor LoF mutant SAMD9 exhibited any inhibitory effect on viral replication (Fig 1D). These results suggest that, similar to the case of HIV-1 [28], SAMD9L, but not SAMD9, inhibits flavivirus replication.

We next investigated whether SAMD9L could inhibit the replication of other flaviviruses, such as DENV and ZIKV. Given the difficulty of infecting HEK293T cells with DENV and ZIKV, we switched to Vero E6 cells for these experiments. Vero E6 cells were transduced with either an empty SCRPSY lentiviral vector or a vector expressing untagged SAMD9L or SHFL, a well-known anti-flavivirus ISG [35,42,43,47]. The expression of these ISGs was confirmed by Western blot (Fig 1E). The cells were then infected with DENV (serotype 2), ZIKV (Asian lineage), WNV (lineage 2), or USUV (Africa 2 lineage) and infection rates were assessed by flow cytometry at 48 or 72 h post-infection (Fig 1F). As expected, overexpression of SHFL reduced replication of all tested viruses (Fig 1F). Interestingly, SAMD9L overexpression exhibited an even more pronounced inhibitory effect, suggesting its potential as a potent and broad-spectrum anti-flavivirus factor (Fig 1F). Similar inhibitory effects were observed for DENV serotypes 3 and 4 (S1 Fig in S1 File).

### Human SAMD9L is a key mediator of the antiviral effects of IFN in myeloid cells

Myeloid cells, including monocytes, macrophages, dendritic cells and microglial cells, are primary targets for flaviviruses and play a critical role in flavivirus-induced pathogenesis [30,48,49]. To evaluate the potential role of SAMD9L in IFN-mediated control of flavivirus propagation, we first assessed its expression in primary human myeloid cells treated with IFN-I.

**Fig 2:**
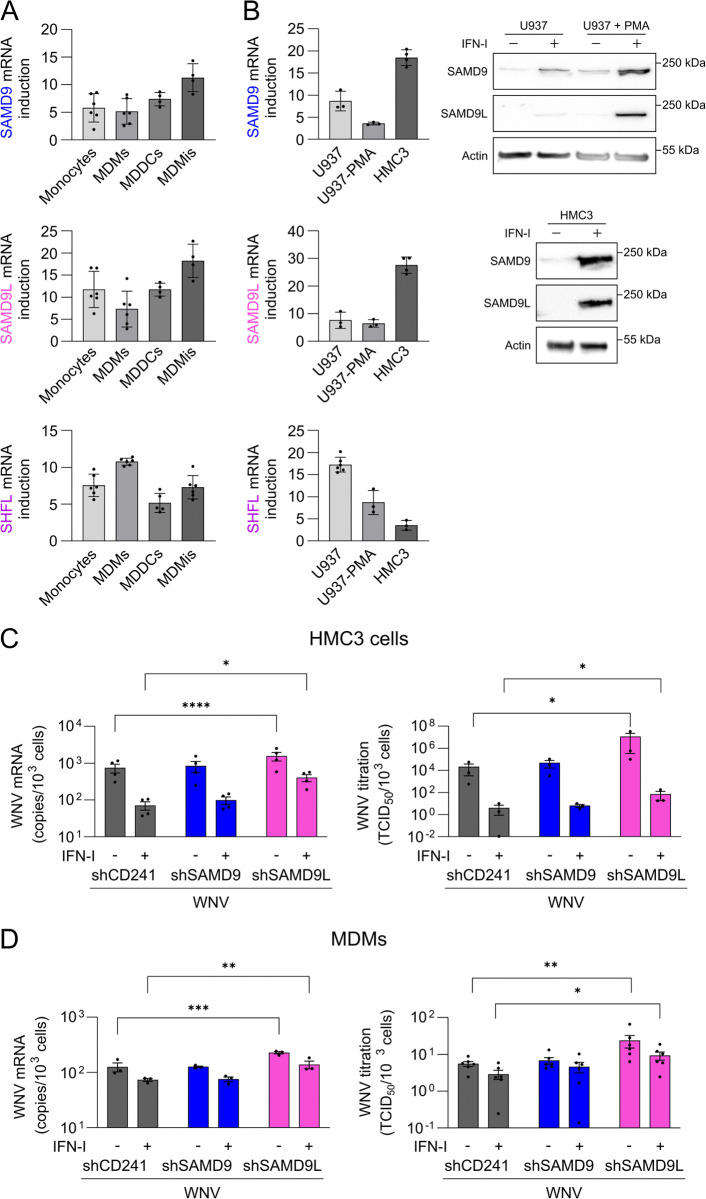
SAMD9L is a key mediator of type I IFN–induced antiviral activity against WNV in myeloid cells. **(A)** Quantification of SAMD9, SAMD9L, and SHFL mRNA induction following 24-hour treatment with IFN-I in various human primary myeloid cell types. Gene expression is shown relative to untreated controls. **(B)** Quantification of SAMD9, SAMD9L, and SHFL induction following 24-hour treatment with IFN-I in human myeloid cell lines (U937 cells either untreated or following PMA-induced differentiation into macrophage-like cells, and HMC3 microglial cells), by RT-qPCR (left) and Western blot (right). **(C)** HMC3 cells were transduced with shRNA-expressing lentiviral vectors for 24 h, treated with IFN-I for 5 h, and then infected with WNV at an MOI of 10 for 24 h. WNV replication was assessed by quantification of viral RNA at 24 h post-infection (RT-qPCR) (left) and by titration of viral particles in supernatants at 48 h post-infection (right). **(D)** Human primary monocyte-derived macrophages (MDMs) were transduced for 24 h, treated with IFN-I for 5 h, and then infected with WNV at an MOI of 10 for 24 h. WNV replication was assessed by quantification of viral RNA (RT-qPCR) (left) and titration of viral particles in supernatants (right). Each dot represents a biological replicate for cell lines or a distinct donor for primary cells. Data are presented as mean ± SEM. Statistical analyses: two-way ANOVA with Fisher’s multiple comparisons test **(C, D)**. Data were log-transformed before analysis (**C** right, **D** right). Statistical significance: ****, p ≤ 0.0001; ***, p ≤ 0.001; **, p ≤ 0.01; *, p ≤ 0.05. Abbreviations: PMA, phorbol 12-myristate 13-acetate; IFN-I, type I interferon; NT, non-transduced.

To this end, monocytes, monocyte-derived macrophages (MDMs), monocyte-derived dendritic cells (MDDCs), and monocyte-derived microglia-like cells (MDMis) were treated with 1000 IU/mL of IFN-I for 24 h or left untreated. SAMD9 and SAMD9L expression levels were then assessed by RT-qPCR, using SHFL [42,43] as an IFN-responsive control gene (Fig 2A). IFN-I treatment induced robust upregulation of SHFL, SAMD9L, and SAMD9 across all tested cell types. SAMD9 and SAMD9L showed similar induction profiles, with induction levels ranging from a 10-fold increase in MDMs and MDDCs to a 20-fold increase in MDMis.

To further validate IFN-I-mediated induction of SAMD9 and SAMD9L, we also examined their expression in relevant human myeloid cell lines, including the monocytic U937 cell line, both untreated (monocyte-like) and following PMA-induced differentiation into macrophage-like cells, as well as the microglial HMC3 cell line (Fig 2B). In these models, SHFL expression was predominantly induced in undifferentiated U937 cells, whereas SAMD9 and SAMD9L expression was most strongly upregulated in HMC3 cells, as confirmed by RT-qPCR and Western blot analysis (Fig 2B).

To investigate the physiological antiviral role of SAMD9L in IFN-mediated resistance to flavivirus infection in myeloid cells, we conducted knockdown experiments targeting endogenous IFN-induced SAMD9L. We chose to perform these experiments in HMC3 cells because both SAMD9L and SAMD9 are strongly induced by IFN-I in these cells (Fig 2B) and they are particularly relevant in the context of flavivirus infections, especially neurotropic flaviviruses like WNV [29,50,51]. Thus, HMC3 cells were transduced with lentiviral particles encoding shRNAs targeting SAMD9 or SAMD9L, or a control shRNA targeting CD241. Twenty-four hours post-transduction, cells were treated with IFN-I for 5 h to induce ISG expression. The efficacy of IFN induction and shRNA-mediated knockdown of SAMD9 and SAMD9L was confirmed by RT-qPCR (S2A Fig in S1 File) and Western blot analysis (S2B Fig in S1 File). Subsequently, cells were infected with WNV for 24 h, and viral RNA levels were quantified by RT-qPCR. As expected, IFN-I treatment strongly suppressed WNV infection, whereas SAMD9L knockdown partially reversed this antiviral effect (Fig 2C, left), supporting the role of SAMD9L as a key ISG that controls flavivirus replication. Interestingly, even in the absence of exogenous IFN-I, SAMD9L knockdown resulted in enhanced WNV replication, suggesting that endogenously produced IFN-I during infection induces sufficient SAMD9L expression to exert partial antiviral effects (Fig 2C, left). In contrast, SAMD9 knockdown did not rescue WNV infection in IFN-treated cells (Fig 2C, left), further confirming that, unlike SAMD9L, SAMD9 does not function as an anti-flavivirus ISG. These results were confirmed by virus titration in supernatants at 48 h post-infection (Fig 2C, right).

To validate these results in another physiologically relevant model, we repeated the experiments in human primary monocyte-derived macrophages (MDMs, S3 Fig in S1 File). Efficient knockdown of SAMD9 and SAMD9L was confirmed by RT-qPCR (S4 Fig in S1 File). Consistent with the observations in HMC3 cells, SAMD9L silencing in primary macrophages partially attenuated the IFN-I–mediated antiviral effect and increased WNV replication even in the absence of IFN-I pretreatment, as shown by RT-qPCR (Fig 2D, left) and corroborated by viral titration assays (Fig 2D, right).

### Human SAMD9L inhibits flavivirus translation

**Fig 3:**
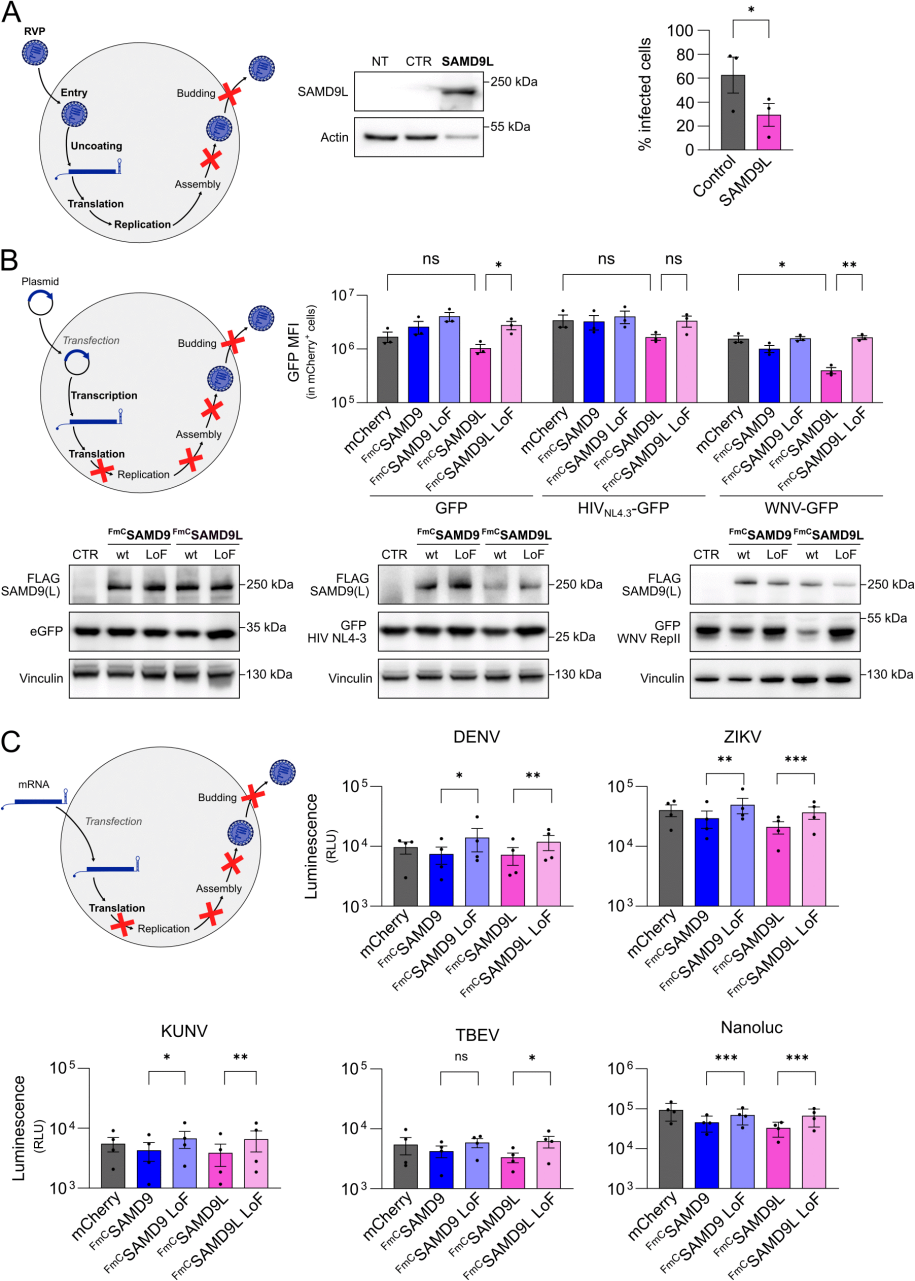
SAMD9L inhibits flavivirus replication by targeting the translation step. **(A)** HEK293T cells were transduced with a lentiviral vector encoding untagged SAMD9L. Expression was confirmed by Western blot. Cells were subsequently infected with GFP-expressing WNV reporter viral particles (RVPs) for 48 h. Transduction efficiency and infection rates were analyzed by flow cytometry. Infection levels are shown in transfected (mCherry^+^) cells relative to the non-transfected (mCherry^-^) cells of the same well (internal control). **(B)** HEK293T cells were co-transfected with either mCherry (control), or N-terminally FLAG-mCherry-tagged (FmC) wt SAMD9, SAMD9 LoF, wt SAMD9L, or SAMD9L LoF expression plasmids, along with constructs encoding GFP in different genetic contexts: under a CMV promoter (CMV-GFP), within an HIV-1 proviral construct (pBR-NL43-IRES-eGFP nef env*), or in a WNV replicon (WNVII Rep-G/Z). GFP fluorescence was quantified by flow cytometry to assess translation efficiency. At the top, cells were analyzed 48 h post-transfection by flow cytometry, gating on mCherry^+^ cells to select for transfected populations. At the bottom, GFP and SAMD9/SAMD9L expression were analyzed by Western blot 48 h post-transfection. **(C)** HEK293T cells were first transfected with the indicated N-terminally FLAG-mCherry-tagged (FmC) SAMD9 or SAMD9L constructs for 48 h, followed by transfection with *in vitro*–transcribed NanoLuc-encoding RNA derived from DENV, ZIKV, WNV Kunjin strain, or TBEV replicons, or from a control standalone plasmid. Eight hours after RNA transfection, translation efficiency was measured by NanoLuc assay. Each dot represents a biological replicate. Data are presented as mean ± SEM. Statistical analyses: paired t-test (A), two-way ANOVA with Fisher’s multiple comparisons test (B), and one-way ANOVA with Fisher’s multiple comparisons test (C). Data were log-transformed prior to analysis (C). Statistical significance: ***, p ≤ 0.001; **, p ≤ 0.01; *, p ≤ 0.05; ns, p > 0.05. The raw data and corresponding calculations used to generate panels A, B, and C are available in S1 Table. Abbreviations: RVPs, reporter viral particles; NT, non-transduced; MFI, mean fluorescence intensity. Schematics were created with Inkscape.

To elucidate the mechanism by which SAMD9L inhibits flavivirus replication, we performed a series of experiments to pinpoint the specific stage of the viral life cycle affected. To distinguish whether the inhibition occurs during an early stage (entry, translation and RNA replication) or a later stage (assembly and budding), we utilized reporter viral particles (RVPs) containing a WNV-based replicon genome. In these replicons, the structural protein-coding region was replaced with an eGFP reporter (S5A Fig in S1 File), enabling infection and RNA replication to be tracked by eGFP expression, while preventing viral assembly and release [52]. RVPs were generated by co-expressing the replicon genome with WNV structural proteins and were then used to infect HEK293T cells, previously transduced with either an empty lentiviral vector or a vector expressing SAMD9L. As shown in Fig 3A, eGFP expression was markedly reduced in SAMD9L-overexpressing cells compared to those transduced with the empty lentivector (Fig 3A), indicating that SAMD9L inhibits flavivirus replication at a stage prior to assembly.

To further pinpoint the stage of the viral cycle affected by SAMD9L, we bypassed the entry step by directly transfecting a GFP-encoding construct, either as a standalone plasmid (CMV-GFP), in the context of an HIV-1 proviral construct (pBR-NL43-IRES-eGFP nef env*), or within a WNV replicon (WNVII Rep-G/Z) (S5A Fig in S1 File). These constructs were transfected in cells overexpressing N-terminally FLAG-mCherry-tagged (FmC) wt or LoF SAMD9, wt or LoF SAMD9L, or mCherry as a control. GFP expression was measured 48 h post-transfection. RT-qPCR analysis confirmed that GFP mRNA levels were comparable across all conditions (S5B Fig in S1 File), indicating that differences in expression were not due to transcriptional effects. In addition, flow cytometry analysis verified that mCherry expression levels were similar across all conditions (S5C Fig in S1 File), ensuring comparable transfection efficiency. As expected, GFP expression was reduced in cells overexpressing wt SAMD9L in all contexts, reflecting its broad inhibitory effect on the translation machinery (Fig 3B). However, this reduction was particularly strong when GFP was expressed within the WNV replicon, as revealed by flow cytometry (Fig 3B, top) and Western blot (Fig 3B, bottom). In contrast, GFP expression from the CMV-GFP plasmid or the HIV-1 NL4.3 IRES-eGFP construct exhibited only a modest, statistically non-significant decrease, indicating that SAMD9L does not measurably inhibit translation of these transcripts. This suggests that the robust inhibition observed with the WNV replicon relies on features intrinsic to flaviviral RNAs rather than reflecting a general block of cellular translation. Collectively, these findings indicate that, as observed with poxviruses [8] and lentiviruses [28], SAMD9L inhibits flavivirus replication at the stage of viral RNA translation. Conversely, GFP expression remained unaffected in cells expressing either wt or LoF SAMD9, or LoF SAMD9L (Fig 3B), consistent with the specificity of SAMD9L-mediated translation inhibition.

Finally, to confirm that translation is the primary stage of the flavivirus life cycle targeted by SAMD9L, we transfected HEK293T cells expressing either wt or LoF variants of SAMD9 or SAMD9L (S6A Fig in S1 File) with *in vitro*–transcribed RNAs encoding NanoLuc, either encoded by flavivirus replicons (DENV, ZIKV, WNV or TBEV) or from a standalone NanoLuc construct (S6B, S6C Fig in S1 File). In this system, NanoLuc expression relies solely on RNA translation, enabling a direct assessment of SAMD9L’s effect on viral RNA translation (Fig 3C). Overexpression of wt SAMD9L markedly inhibited NanoLuc expression from all flavivirus replicons compared to its LoF mutant (Fig 3C), confirming that SAMD9L restricts flavivirus replication primarily by targeting the translation step. These results also highlight the essential role of its Schlafen-like ribonuclease domain in mediating this antiviral activity. Interestingly, wt SAMD9 also suppressed translation from the replicons and control RNA, despite lacking antiviral activity during active infection. This result strongly suggests that SAMD9 is intrinsically capable of inhibiting flaviviral RNA translation when the context of infection is bypassed, suggesting that its activation may be virus-specific or that flaviviruses have evolved mechanisms to evade or counteract SAMD9’s antiviral activity (Fig 3C).

### Human SAMD9L restricts flavivirus infection independently of innate immune activation

**Fig 4:**
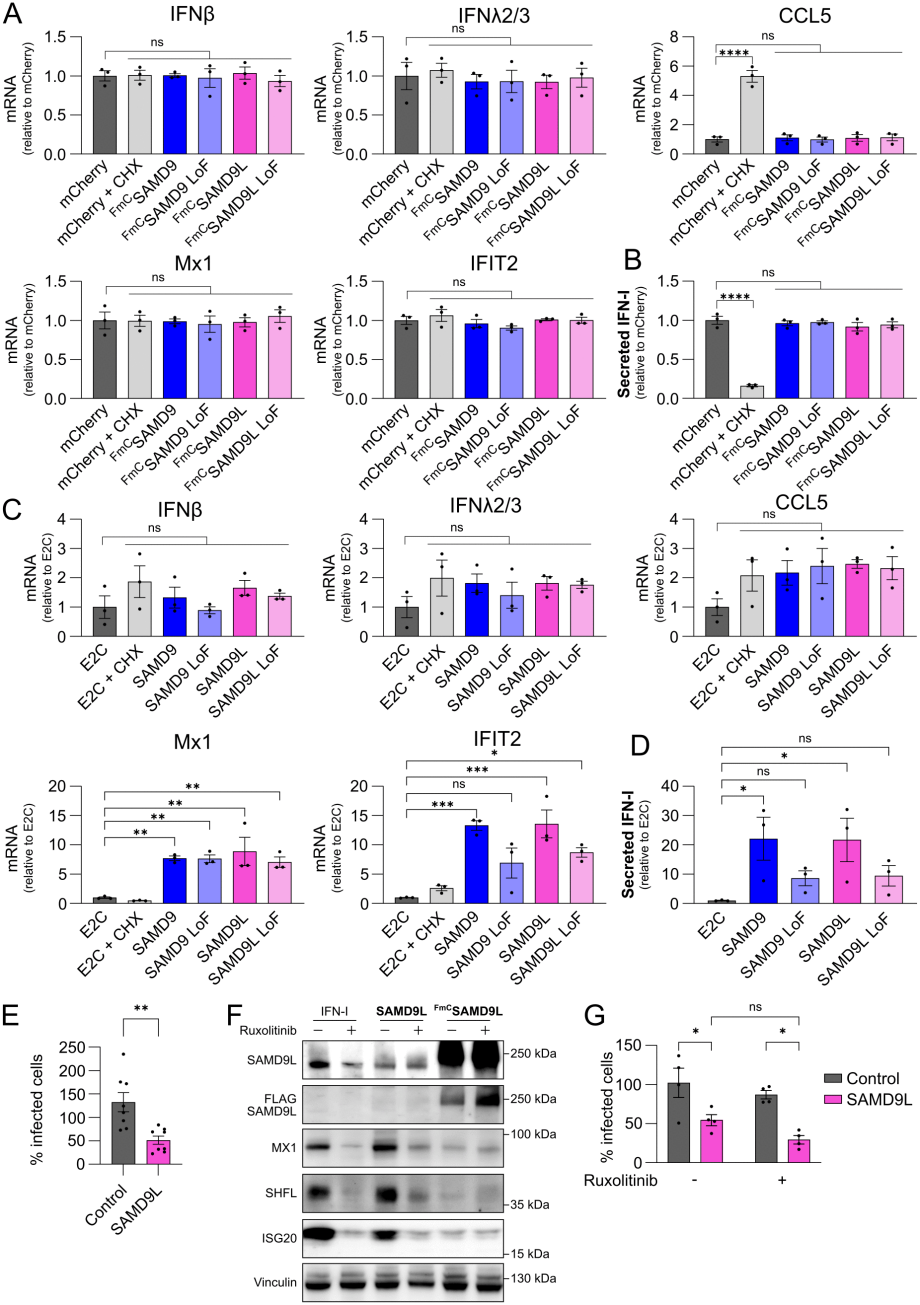
SAMD9L-mediated innate immune activation and flavivirus restriction are independent mechanisms. **(A)** A549 cells were transfected with plasmids expressing N-terminally FLAG-mCherry-tagged (FmC) SAMD9 or SAMD9L (wt or LoF) for 24 h. Cells were treated or not with 10 µg/mL of CHX 8 h before cell lysis. Expression of IFNs, ISGs, and chemokines was quantified by RT-qPCR. **(B)** IFN-I secretion was measured in supernatants from the same transfected cells using a luciferase-based assay at 48 h post-transfection. **(C)** A549 cells were transfected with plasmids expressing untagged SAMD9 or SAMD9L (wt or LoF) for 24 h. Cells were treated or not with 10 µg/mL of CHX 8 h before cell lysis. Expression of IFNs, ISGs, and chemokines was quantified by RT-qPCR. **(D)** IFN-I levels in the supernatants of transduced cells were assessed using a luciferase-based assay at 48 h post-transfection. **(E)** HEK293T cells were transduced with a lentiviral vector encoding untagged SAMD9L and subsequently infected with WNV (MOI 1) for 48 h. Transduction efficiency and infection rates were determined by flow cytometry. Infection levels are expressed as the percentage of infected cells among the transduced population (TagRFP^+^), normalized to non-transduced cells (TagRFP^-^) within the same well (internal control). **(F)** A549 cells were transfected with plasmids expressing N-terminally FLAG–mCherry–tagged (FmC) SAMD9L, untagged SAMD9L, or treated with 100 U/mL IFN-I for 24 h. SAMD9L expression and a panel of ISGs (Mx1, SHFL, and ISG20) were analyzed by Western blot. **(G)** A549 cells were transduced with a lentiviral vector encoding untagged SAMD9L and treated 6 h post-transduction with Ruxolitinib (10 µM) or DMSO (vehicle control), followed by WNV infection (MOI 1) for 48 h. Transduction efficiency and infection rates were analyzed by flow cytometry. Infection levels are expressed as the percentage of infected cells among the transduced population (TagRFP^+^), normalized to non-transduced cells (TagRFP^-^) within the same well (internal control). Each dot represents a biological replicate. Data are presented as mean ± SEM. Statistical analyses: one-way ANOVA with Dunnett’s multiple comparison test **(A–D)**, unpaired t-test **(E)**, and two-way ANOVA with Tukey’s multiple comparison test **(G)**. Statistical significance: ****, p ≤ 0.0001; ***, p ≤ 0.001; **, p ≤ 0.01; *, p ≤ 0.05; ns, p > 0.5. Abbreviations: CHX, cycloheximide; E2C, E2 Crimson; IFN-I, type I interferon.

Human SAMD9 was found to trigger antiviral signaling when overexpressed, as measured by ISRE reporter activation in a screening of interferon-stimulated genes [53], and has been recently identified as a cytoplasmic PRR that senses double-stranded nucleic acids and activates type I IFNs, cytokines, and chemokines [27]. In the same study, Hou et al. showed that this PRR-like activity is also conserved in various homologs of human SAMD9: human, mouse and hamster SAMD9Ls, as well as a zebrafish SAMD9L homolog, all of which were able to trigger innate immune gene expression upon overexpression [27]. These findings point to a conserved role for SAMD9/9L gene family as antiviral sensors across vertebrate species. In order to assess whether human SAMD9L’s anti-flavivirus activity stems from a direct inhibition of viral translation or is instead mediated by IFN induction and the expression of other antiviral ISGs, we transfected A549 cells with N-terminally FLAG-mCherry-tagged wt or LoF mutants of SAMD9 or SAMD9L. We then quantified the expression of transcripts previously shown to be upregulated by SAMD9 overexpression (i.e., IFN-β, IFN-λ2/3, CCL5, CXCL10, Mx1, and IFIT2), as reported by Hou et al [27]. As a control, cells were treated with cycloheximide to block translation independently of SAMD9/SAMD9L expression. Surprisingly, overexpression of SAMD9 and SAMD9L from our constructs, whether wt or LoF, did not lead to increased expression of IFN, ISGs or chemokines at 24 h (Figs 4A and S7A in S1 File), nor did it result in detectable secretion of IFN-I at 48h post-transfection (Fig 4B). A key difference between our constructs and those used in the study by Hou et al. is the tag position. Indeed, the pMT06 plasmids used in our study express N-terminally tagged SAMD9 and SAMD9L, like in most studies [8,12,25,28], whereas Hou et al. used C-terminally tagged constructs [27]. To assess whether the tag position could affect SAMD9L’s ability to induce innate immune responses, we repeated the experiment using untagged wt of LoF mutants or SAMD9 or SAMD9L (Figs 4C and S7B in S1 File). At 24 h post-transfection, all constructs significantly upregulated the expression of Mx1 and IFIT2 (Fig 4C), while only wt SAMD9 and SAMD9L induced high levels of secreted IFN-I at 48 h post-transfection (Fig 4D). Of note, that suggests that the SLFN-like catalytic domain might play a role also in the sensor roles of SAMD9 and SAMD9L. We further confirmed these results using the SCRPSY lentiviral vector encoding an untagged version of SAMD9L that we used to transduce A549 cells. Forty-eight hours post-transduction, RT-qPCR analysis revealed a modest but consistent upregulation (1.5- to 3-fold) of IFN, ISG, and chemokine transcripts (S7C Fig in S1 File), accompanied by a corresponding increase in secreted IFN-I levels (S7D Fig in S1 File). Together, these results confirm that SAMD9L overexpression is sufficient to trigger an innate immune response. However, the ability of our N-terminally tagged SAMD9L constructs to potently inhibit flavivirus replication and translation without detectable immune activation suggests that these two activities are likely mechanistically independent. This conclusion is further supported by experiments performed in HEK293T cells, which are largely deficient in IFN-I signaling. In this context, neither the tagged nor the untagged versions of SAMD9 or SAMD9L induced detectable IFNs, ISGs, or chemokines (S8 Fig in S1 File). Since we showed that SAMD9L inhibits WNV and USUV replication in these cells (Fig 1A and 1D), these findings support the hypothesis that SAMD9L’s antiviral activity is independent of innate immune activation. We confirmed that untagged SAMD9L inhibits WNV replication in HEK293T cells (Fig 4E) and that it inhibits GFP translation in the context of a WNV replicon (S9 Fig in S1 File) as efficiently as the N-terminally tagged protein (Fig 3B), demonstrating that its antiviral activity is intrinsic and not affected by tagging.

To confirm that SAMD9L’s anti-flaviviral activity is independent of its ability to induce an innate immune response, we used Ruxolitinib, a potent and selective JAK1/JAK2 inhibitor that blocks type I interferon signaling and downstream ISG induction [54] (S10 Fig in S1 File). As shown by Western blot, treatment with IFN or overexpression of untagged SAMD9L induced expression of the ISGs Mx1, SHFL, and ISG20, and this induction was efficiently suppressed by Ruxolitinib (Fig 4F). In contrast, as expected, the N-terminally tagged SAMD9L (FmC) failed to induce ISG expression (Fig 4F). We then transfected untagged SAMD9L into A549 cells treated or not with Ruxolitinib and infected them with WNV, followed by flow cytometry analysis of infection rates. As shown in Fig 4G, untagged SAMD9L inhibited WNV replication regardless of whether IFN signaling and ISG induction were blocked by Ruxolitinib. Together, these results demonstrate that SAMD9L inhibits flavivirus replication by suppressing viral RNA translation, independently of its capacity to activate an antiviral innate immune response.

## Discussion

SAMD9 and its paralog SAMD9L are emerging as major antiviral effectors in cellular defense. Their role is well established in the case of poxviruses, where antiviral activity of both human SAMD9/9L and murine SAMD9L has been demonstrated [8,15,25,26]. In addition, human SAMD9L has been shown to restrict the replication of HIV-1 and other lentiviruses [28]. More recently, human SAMD9 and murine SAMD9L have also been shown to inhibit the replication of reoviruses and rotaviruses [27]. The identification of structural homologs of SAMD9L in bacterial defense systems [10] further supports its broader role in antiviral immunity. In this study, we further extend the known antiviral spectrum of human SAMD9L by demonstrating its ability, unlike SAMD9, to inhibit the replication of flaviviruses. Specifically, SAMD9L overexpression significantly suppressed replication of all flaviviruses tested (WNV, ZIKV, DENV and USUV). Conversely, knockdown of SAMD9L in human microglial cells and primary macrophages partially abrogated the antiviral activity of IFN-I against WNV, whereas silencing SAMD9 had no effect. This finding underscores the critical role of SAMD9L in IFN-I-mediated defense, particularly in myeloid cells, which are major targets of flaviviruses and central to flavivirus-induced pathogenesis. Interestingly, SAMD9L knockdown also led to increased WNV replication even in the absence of exogenous IFN-I, suggesting that infection-induced, endogenous IFN-I is sufficient to upregulate SAMD9L expression to levels that can restrict WNV replication. Further mechanistic analysis revealed that SAMD9L targets the viral RNA translation stage of the flavivirus replication cycle, consistent with previous reports that both SAMD9 and SAMD9L function as potent inhibitors of translation [8,12,28,55]. Our results further demonstrate that the efficiency of SAMD9L-mediated translational inhibition is modulated by the RNA context. Specifically, when GFP is expressed from a plasmid or an HIV-1-derived construct based on the NL4.3 strain, SAMD9L’s inhibitory effect was modest and not statistically significant, consistent with reports that HIV-1 sensitivity to SAMD9L is strain-dependent [28]. In contrast, GFP translation was more efficiently blocked when encoded within a WNV replicon, indicating that features specific to viral RNAs may condition SAMD9L responsiveness. Flaviviral RNAs possess a distinct combination of structural and sequence elements, such as highly structured 5’ and 3’ untranslated regions, conserved internal RNA motifs, and long-range interactions involved in genome cyclization, any of which could serve as recognition or activation signals for SAMD9L. Although the precise molecular determinants remain unknown, these features may promote selective translational repression by facilitating SAMD9L recruitment or activation on viral transcripts. Consistent with this model, the LoF mutation in the Schlafen-like ribonuclease domain completely abrogated antiviral activity, supporting a mechanism in which SAMD9L directly engages structured viral RNAs through its catalytic domain rather than acting via non-specific nucleic acid degradation. Collectively, these findings provide a plausible basis for the preferential targeting of flaviviral transcripts in our system.

Although the precise molecular mechanisms by which SAMD9 and SAMD9L inhibit cellular and viral translation remained unclear for a long time, the recent discovery that human SAMD9 possesses anticodon nuclease activity (ACNase), specifically cleaving phenylalanine tRNAs (tRNA^Phe^), has significantly advanced our understanding of the global translational arrest mediated by these proteins [8]. Interestingly, targeted tRNA cleavage as an antiviral strategy is notably employed by another well-known antiviral factor called Schlafen 11 (SLFN11), which inhibits the translation and replication of HIV-1 [6], as well as other viruses, including WNV, DENV, and ZIKV [44]. Other members of the Schlafen protein family also exhibit antiviral activity, linked to their nucleic acid-binding properties and endoribonuclease function, which is conferred by a conserved Schlafen-box, also known as the Schlafen core domain [56–58]. Interestingly, Legrand et al. recently identified a conserved Schlafen-like ribonuclease motif within the AlbA2 domain of mammalian SAMD9/9L and showed that specific mutations in this catalytic site abolish SAMD9L-mediated inhibition of HIV-1 translation [28]. Using the same LoF mutant, we further demonstrated that the ability of SAMD9L to inhibit flavivirus replication also depends on this ribonuclease domain. The fact that SAMD9, which cleaves tRNA^Phe^ via this domain [12], does not inhibit retrovirus or flavivirus replication, suggested that SAMD9L targets a different RNA species. However, we found that SAMD9 can inhibit the translation of viral RNA when directly transfected into cells, indicating that it has the inherent capacity to block flavivirus RNA translation outside the context of infection. This observation implies that the distinct specificities of SAMD9 and SAMD9L may arise from differences in how they recognize or become activated by viral RNA, rather than from the RNA species they target. Supporting this idea, SAMD9’s ACNase activity was recently shown to be triggered by poxvirus infection [8], suggesting it may remain inactive during flavivirus replication. Alternatively, flaviviruses may encode proteins that inhibit SAMD9 (but not SAMD9L) function.

The ability of SAMD9 and SAMD9L to detect and respond to viral RNA indicates that these proteins could function both as viral sensors and antiviral effectors [8]. This dual function is further supported by recent findings showing that SAMD9, but also SAMD9L, from multiple species can detect cytosolic double-stranded nucleic acids and initiate innate immune signaling, acting as cytoplasmic PRRs [27]. To verify whether SAMD9L can activate the innate immune response upon overexpression, which could account for its antiviral activity, we transfected A549 cells with plasmids encoding either SAMD9 or SAMD9L and measured the expression of innate immune transcripts by RT-qPCR, following the approach described by Hou et al. However, we did not observe any induction of antiviral gene expression at 24 h post-transfection with either construct. One important difference between our study and that of Hou et al. lies in the tagging strategy. While our constructs express N-terminally tagged proteins, as is the case in most studies on SAMD9/9L [8,12,25,28], their constructs carry a C-terminal tag [27]. To assess whether tag position could influence our results, we repeated the experiments using untagged wt or LoF SAMD9 and SAMD9L. These experiments showed that untagged SAMD9L can elicit a modest but significant innate immune response, inducing IFN and ISG expression, consistent with previous observations [27]. However, this activity is unlikely to represent the primary mechanism underlying its antiviral effect against flaviviruses. Indeed, most of our experiments were performed with an N-terminally tagged version of SAMD9L, which robustly inhibits flavivirus replication and translation without triggering antiviral gene expression. Moreover, SAMD9L maintained its antiviral activity in cells deficient in type I IFN responses, such as HEK293T and Vero cells [59,60]. Finally, treatment of IFN-competent A549 cells with the JAK1/JAK2 inhibitor Ruxolitinib confirmed that SAMD9L’s antiviral activity against WNV is preserved even when IFN signaling and ISG induction are blocked, demonstrating that human SAMD9L restricts flavivirus replication primarily through direct inhibition of viral RNA translation, independently of innate immune activation. We noted, however, that N-terminally tagged SAMD9L accumulates to higher steady-state levels than its untagged counterpart (Fig 4F). Such accumulation could, in principle, enhance non-specific translational repression through stress-granule formation once intracellular concentrations exceed a certain threshold. Nonetheless, several lines of evidence argue against this being the main driver of the antiviral phenotype. First, despite its higher accumulation, the N-terminally tagged SAMD9L inhibits flavivirus translation without triggering detectable IFN induction, demonstrating that translational repression can occur independently of innate immune activation. Second, inhibition remains strictly dependent on an intact Schlafen-like catalytic domain, indicating that the antiviral effect reflects specific enzymatic activity rather than a global stress-granule–mediated shutdown. Third, the antiviral activity of untagged SAMD9L is largely comparable to the tagged version despite its lower expression levels. While these observations support a functional separation between SAMD9L’s antiviral and innate immune activities, we acknowledge that differences in protein abundance may modulate the magnitude of the effect. Future studies using endogenous tagging or conditional expression systems will be required to delineate how expression levels and additional factors influence SAMD9L antiviral activity.

SAMD9 and SAMD9L are emerging as antiviral factors capable of both sensing and restricting a broad spectrum of viruses, placing them among a growing class of dual sensor-effector ISGs, such as PKR (EIF2AK2) [61], IFIT1 [62] or TRIM5α [63,64]. Together with recent studies, our findings support a dual role for these proteins as cytoplasmic sensors and translation inhibitors, while also revealing functional divergence between the paralogs despite their protein structural similarity [9–12]. These distinctive features open promising avenues for future research into the mechanisms by which SAMD9 and SAMD9L recognize viral RNA and orchestrate antiviral defense.

## Materials and methods

### Cell culture

HEK293T (CRL-11268), Vero E6 (CRL-1586), HMC3 (CRL-3304), A549 (CCL-185) and C6/36 cells (CRL-1660) were purchased from the American Type Culture Collection (ATCC). STING-37 cells were kindly provided by P.O. Vidalain (CIRI, Lyon, France) [65]. All cell lines, except C6/36 and HMC3, were cultured in complete high-glucose Dulbecco′s modified Eagle′s medium (DMEM, Gibco) supplemented with 10% fetal bovine serum (FBS, Serana), and 1% Penicillin/Streptomycin (Gibco). HMC3 cells were cultured in complete high-glucose Dulbecco′s modified Eagle′s medium (DMEM, Gibco) supplemented with 10% fetal bovine serum (FBS, Serana), 1% Penicillin/Streptomycin (Gibco) and 1% non-essential amino acids (NEAA, Gibco). Mammalian cells were kept at 37°C with 5% CO_2_. *Aedes albopictus* C6/36 cells were cultured in Leibovitz′s L15 medium (Gibco) supplemented with 10% FBS, 1% Penicillin/Streptomycin, 1% NEAA and 1% tryptose phosphate broth (Gibco), and were kept at 28°C without CO_2_.

Buffy coats from healthy donors were obtained from the Etablissement Français du Sang (EFS, Montpellier, France). PBMCs were isolated by density centrifugation using Lymphoprep medium (STEMCELL Technologies) and cultured in Roswell Park Memorial Institute (RPMI) 1640 medium (Gibco) supplemented with 10% FBS and 1% Penicillin/Streptomycin. Monocytes were isolated from PBMCs by plastic adhesion for 45 min and then differentiated into monocyte-derived macrophages (MDMs), monocyte-derived dendritic cells (MDDCs), or monocyte-derived microglia-like cells (MDMis). MDMs were differentiated in culture medium supplemented with 50 ng/mL GM-CSF (Gentaur) for 8–10 days. MDDCs were differentiated in culture medium supplemented with 100 ng/mL IL-4 (CytoBox Mo-DC, Miltenyi) and 50 ng/mL GM-CSF for 7 days. MDMis were differentiated in culture medium supplemented with 10 ng/mL GM-CSF, 10 ng/mL M-CSF (PeproTech), 100 ng/mL CCL2 (PeproTech), 10 ng/mL NGF-β (PeproTech), and 100 ng/mL IL-34 (PeproTech) for 10 days.

### Viruses

WNV lineage 2 (WNV-6125/France/2018) and USUV Africa 2 (Rhône2705/France/2015) were provided by ANSES (National Agency for Food, Environmental and Occupational Health Safety, France). DENV-2 (UVE/DENV-2/2014/FR/CNR_26104) and ZIKV (strain H/PF/2013) were kindly provided by Xavier de Lamballerie (Unité des Virus Emergents, Aix Marseille Université, Marseille, France), through the European Virus Archive GLOBAL (EVA-GLOBAL) project. All viral stocks were prepared by infecting C6/36 cells for 2 h at a low multiplicity of infection (MOI). After infection, the inoculum was removed, fresh complete L-15 medium was added, and supernatants were collected at 5 (WNV and USUV), 7 (DENV), or 10 (ZIKV) days post-infection. Viral titers were determined by TCID_50_ using the Spearman–Kärber method on A549 cells (WNV and USUV) and flow cytometry on Vero E6 cells (DENV and ZIKV).

Infections of HEK293T, A549 and Vero E6 cells were carried out by inoculating cells with a low volume of viral dilution in serum-free DMEM for 2 h, at an appropriate MOI as detailed in Fig legends. The inoculum was then removed and replaced with fresh DMEM, 2% FBS, 1% Penicillin/ Streptomycin. For HMC3 cells and MDMs, the viral dilution was done in DMEM, 2% FBS, 1% NEAA, 1% Penicillin/Streptomycin and RPMI, 2% FBS, 1% Penicillin/Streptomycin, respectively.

### Plasmid constructs and gene exogenous expression

Untagged human SHFL and SAMD9L cloned under a CMV promoter in lentiviral constructions from the SCRPSY library (GenBank accession no. KT368137) that express a red fluorescent protein (TagRFP) and puromycin resistance [29,66], were kindly provided by Sam J. Wilson (MRC-University of Glasgow Centre for Virus Research, Glasgow, UK). An empty vector in the same backbone (SCRPSY) was used as a control. To produce lentiviral particles, lentiviral constructs were co-transfected with expression plasmids of VSV-G (pMD2.G, Addgene accession no. 12259), and HIV-1 gag-pol (pCMVR8.74, Addgene accession no. 22036) in HEK293T cells using polyethylenimine (PEI, Polysciences). Culture medium was replaced 8 h post-transfection and supernatants were collected 48 h post-transfection. Lentiviral titers were determined by flow cytometry. Transduction was performed using a lentiviral particle dose that yielded approximately 50 percent transduction efficiency, with spinoculation at 1000 × *g* for 10 min.

Overexpression of mCherry, and N-terminally FLAG-mCherry-tagged SAMD9, SAMD9 D241A (LoF, loss-of-function), SAMD9L and SAMD9L E198A/D243A (LoF) was achieved using plasmids constructs encoding each protein cloned into the pMT06 backbone (a gift from Caroline Goujon: Addgene plasmid #139448 [67]). FLAG-mCherry-SAMD9(L) plasmids were previously described by Legrand and colleagues [28], whereas the remaining constructs were generated de novo for this study. Briefly, the pMT06-FLAG-mCherry-SAMD9L and pMT06-FLAG-mCherry-SAMD9 (synthesized and cloned by Azenta Genewiz) plasmids were used to generate the LoF mutants using the QuikChange Lightning Site-Directed Mutagenesis Kit (Agilent) following the manufacturer’s instructions. Sequences were confirmed through full-length plasmid or Sanger sequencing (Microsynth). Overexpression of E2 Crimson and untagged SAMD9, SAMD9 D241A (LoF), SAMD9L, and SAMD9L E198A/D243A (LoF) was achieved using constructs cloned into the pSFFV backbone (a gift from Caroline Goujon: Addgene plasmid #139445 [67]). Untagged SAMD9 constructs (wt and LoF) were generated by restriction digestion of the corresponding FLAG-mCherry-tagged ORFs in pMT06 (described above), whereas untagged SAMD9L constructs (wild-type and LoF) were amplified by PCR from the pMT06 templates to remove the tag, using the following primers: 5’-AATTGGATCCACCATGGCAAAGCAACTTAACCTTCCAG-3’  and 5’AATTCTCGAGCTAAGCGTAATCTGGAACATCGTATGGGTAAACAATTTCAATGTCATAAGCAAG-3’. PCR products and digested fragments were inserted into pSFFV to replace E2 Crimson by classical restriction digestion and T4 ligation, and sequences were confirmed through full-length plasmid sequencing (Plasmidsaurus). All plasmids were transfected into target cells using Lipofectamine 2000 (Invitrogen) or Fugene 6 (Promega). When indicated, cycloheximide (CHX, Merck) was added at 10 µg/mL for the duration specified in the Fig legends.

Co-transfection of N-terminally FLAG-mCherry-tagged (FmC) or untagged wt SAMD9, SAMD9 LoF, wt SAMD9L, or SAMD9L LoF with CMV-GFP (pcDNA3.1-GFP), WNV replicon construct expressing eGFP (WNVII Rep-G/Z, a kind gift of Theodore Pierson), or HIV-1 proviral construct (pBR-NL43-IRES-eGFP nef env*, previously described in [68]) was achieved with PEI (Polysciences) or Lipofectamine 2000 (Invitrogen), with equal amount of each plasmid. Cells were incubated 48 h prior to analysis. Where indicated, cells were treated with Ruxolitinib (MedChemExpress) at a final concentration of 10 µM for the duration specified in the Fig legends.

### Flow cytometry

For mCherry, TagRFP or eGFP expression quantification, cells were detached with Trypsin-EDTA 0.25% (Gibco) and pelleted for 5 min at 500 × *g*. Cells were washed with PBS, pelleted, and fixed with 4% formaldehyde in PBS (Thermo Fisher Scientific) for 15 min. Cells were then washed with PBS to remove excess of formaldehyde. For quantification of flavivirus infection, infected cells were detached, fixed, and incubated for 2 h at room temperature with the pan-flavivirus anti-Env 4G2 primary antibody (Novus Biologicals) diluted 1/2000 in PBS, 1% bovine serum albumin (BSA, Thermo Fisher Scientific) and 0.05% saponin. Cells were then washed twice with PBS, 1% BSA, 0.05% saponin and incubated for 1 h at room temperature with anti-mouse secondary antibody coupled with Alexa Fluor 488 (Thermo Fisher Scientific) diluted 1/2000 in PBS, 1% BSA and 0.05% saponin. Cells were then washed again with PBS, 1% BSA, 0.05% saponin. Flow cytometry was performed using a Novocyte flow cytometer (Agilent). For primary macrophages phenotyping, cells were detached with PBS supplemented with 5 mM EDTA (Invitrogen) and gentle scraping. Cells were then pelleted, washed and fixed as described above. Staining was performed by incubating the cells for 30 min at 4°C with fluorophore-conjugated antibodies, according to manufacturer’s instructions (Table 1). After staining, cells were washed twice with PBS and analyzed using an LSR Fortessa (BD Biosciences). All flow cytometry analyses were performed with FlowJo v10 for Windows (BD Biosciences).

**Table 1:** Fluorophore-conjugated antibodies used for cytometry.

Target	Fluorescence	Brand	Clone	Reference
CD3	BV421	Biolegend	UCHT1	300433
CD11b	APC/Cy7	Biolegend	M1/70	101225
CD14	PerCP/Cy5.5	Biolegend	HCD14	325621
CD16	Alexa Fluor 700	Biolegend	3G8	302026
HLA-DR	FITC	Biolegend	L243	980402
CD80	BV650	Biolegend	2D10	305227

### Western blot

Cells were washed with PBS, lysed in ice-cold RIPA buffer (50 mM Tris-HCl [pH 8], 150 mM NaCl, 0.5% NP-40, 2mM EDTA) for 15 min with periodic scraping. Samples were heat-denatured at 95°C for 10 min in 4X Laemmli buffer (250 mM Tris-HCl [pH 7], 8% sodium dodecyl sulfate [SDS], 40% glycerol, 10% β-mercaptoethanol, and 0.005% bromophenol blue), underwent SDS-PAGE using SureCast Gel Handcast System (Thermo Fisher Scientific), followed by transfer onto a 0.45 μm nitrocellulose membrane (Amersham). Membranes were blocked in PBST (PBS, 0.05% Tween 20) with 10% fat-free milk (Regilait) for 30 min. Primary antibodies and horseradish peroxidase (HRP) conjugated secondary antibodies (Table 2) were diluted in PBST containing 1% BSA. Protein visualization was achieved by detection of HRP activity (Immobilon Forte Western HRP substrate, Merck) on a ChemiDoc imaging system (Bio-Rad). When necessary, membranes were stripped with Strip buffer (15g/L glycine, 1% SDS, 1% Tween20 in water, buffered at pH 2.2) for 15 min at RT before new staining. Images were analyzed with ImageJ software.

**Table 2:** Antibodies used for Western blot analyses.

Type	Species	Target	Brand	Reference
Primary	Rabbit	SAMD9	Merck	HPA021319
	Rabbit	SAMD9L	Proteintech	25173-1-AP
	Rabbit	SHFL	Proteintech	27865-1-AP
	Mouse	Actin	Merck	A1978
	Rabbit	GFP	Proteintech	PABG1
	Mouse	Vinculin	Proteintech	66305-1
	Mouse	FLAG	Merck	F3165
	Rabbit	Mx1	Thermo Scientific	PA5–22101
	Rabbit	ISG20	Proteintech	22097-1-AP
Secondary (HRP-conjugated)	Donkey	Rabbit IgG	Cytiva	NA934
	Sheep	Mouse IgG	Cytiva	NA931

### Stimulation of ISG expression by interferon

Primary cells and cell lines were cultured in their culture medium with 1000 IU/mL of universal type I interferon (PBL Assay Science) for 24 h to induce the expression of ISGs. Cells were then washed with PBS to remove interferon and used for further analysis.

### Real-time quantitative RT-PCR (RT-qPCR)

Cellular RNAs were extracted using the RNeasy Mini kit (Qiagen) following the manufacturer′s instructions. RNA concentration and purity were evaluated by spectrophotometry (NanoDrop 2000c, Thermo Fisher Scientific). A maximum of 500 ng of RNA was reverse transcribed with both oligo dT and random primers using a PrimeScript RT Reagent Kit (Perfect Real Time, Takara Bio Inc.). Real-time PCR reactions were performed in duplicate using Takyon ROX SYBR MasterMix blue dTTP (Eurogentec) on an Applied Biosystems QuantStudio 5 (Thermo Fisher Scientific) or in triplicate using PowerUp Sybr Green Master Mix (Thermo Fisher Scientific) on an Applied Biosystems Viia 7 (Thermo Fisher Scientific). Cellular transcripts were quantified with primers which hybridize the cDNA sequences (Table 3). Viral genomes were quantified using primers designed to target specific sites within the 3′ untranslated region (Table 3). Plasmids into which WNV amplicons have been cloned were used for absolute quantification of viral cDNA. Quantifications were performed with the following program for Takyon Master Mix: 3 min at 95°C, 40 cycles of 15 s at 95°C, 20 s at 60°C and 20 s at 72°C. The following program was used with PowerUp Master Mix (Thermo Fisher Scientific): 2 min at 50°C, 2 min at 95°C, 45 cycles of 1 s at 95°C and 30s at 60°C. Melting curves were also assessed.

**Table 3:** Sequences of primers used for qPCR analyses.

Target	Direction	Sequence (5’ → 3’)
RPL13A	F	AACAGCTCATGAGGCTACGG
	R	TGGGTCTTGAGGACCTCTGT
SAMD9	F	ACAATACCCATCACTCCCGC
	R	TCATAAGCAAGTGGGCCTCC
SAMD9L	F	TCCATGACAGCCATCGCTAC
	R	TCCGTGGCTGTTTCTGTGTT
SHFL	F	GTATCCTCCAAGAAGGCGGG
	R	TTGCTTTACCCCGTACACGA
WNV	F	AGTTGAGTAGACGGTGCTGC
	R	CTCCTTCCGAGACGGTTCTG
eGFP	F	GAAGGCTACGTCCAGGAG
	R	CGGTTCACCAGGGTGTC
IFN-β	F	TGCTCTCCTGTTGTGCTTCTC
	R	CAAGCCTCCCATTCAATTGCC
IFN-λ2/3	F	TGACCGTGACTGGAGCAGTT
	R	CTAAGGCATCTTTGGCCCTCT
IFIT2	F	AATAGGACACGCTGTGGCTC
	R	AGGCTGGCAAGAATGGAACA
Mx1	F	TCGTACTGGGAAAGGGATTTT
	R	GCTGTGATGTTCAGCATGTGT
CCL5	F	CTGCTTTGCCTACATTGCCC
	R	TCGGGTGACAAAGACGACTG
CXCL10	F	CGCTGTACCTGCATCAGCAT
	R	GCAATGATCTCAACACGTGGAC
ISG20	F	TGACCTGAAGCACGACTTCC
	R	CAAAACAGCCTGTCAGTGGA

### Selective ISG knockdown

Short hairpin RNAs (shRNAs) targeting the transcripts encoding CD241, SAMD9 and SAMD9L were designed using the Broad Institute Genetic Perturbation platform. Corresponding pLKO.1-shRNA plasmids were obtained from Merck. The three shRNA sequences targeting SAMD9L are identical to those previously described in [28]:

5’-CCGGGCAGACAGTATTGCACTAAATCTCGAGATTTAGTGCAATACTGTCTGCTTTTTTG,
5’-CCGGCATCGCTACATAGAACATTATCTCGAGATAATGTTCTATGTAGCGATGTTTTTTG,
5’-CCGGGCTCTTATGTTACTGACTCTACTCGAGTAGAGTCAGTAACATAAGAGCTTTTTTG.

The three shRNA sequences targeting SAMD9 are:

5’-CCGGCTGGGAACTAAGGAAGAAATTCTCGAGAATTTCTTCCTTAGTTCCCAGTTTTTTG
5’-CCGGCCCTCTTACTGATTATGCTTTCTCGAGAAAGCATAATCAGTAAGAGGGTTTTTTG
5’-CCGGCATCGTACAAAGCAACCAATTCTCGAGAATTGGTTGCTTTGTACGATGTTTTTTG

Control shRNA sequences targeting CD241 (also named RHAG) are as described in [69]:

5’-CCGGGCAAGAATAGATGTGAGAAATCTCGAGATTTCTCACATCTATTCTTGCTTTTTG,
5’-CCGGCCTCTGACATTGGAGCATCAACTCGAGTTGATGCTCCAATGTCAGAGGTTTTTG,
5’-CCGGGATGACAGGTTTAATTCTAAACTCGAGTTTAGAATTAAACCTGTCATCTTTTTTG.

To produce lentivectors, constructions carrying shRNAs were co-transfected with VSV-G (pMD2.G, Addgene accession no. 12259), and HIV-1 gag-pol (pCMVR8.74, Addgene accession no. 22036) in HEK293T cells using calcium chloride co-precipitation method. Culture medium was replaced 8 h post-transfection and supernatants were collected 48 h post-transfection. Lentiviral particle titers were determined by quantification of p24 protein with Lenti-X-P24 rapid titer kit (Takara). Cells were transduced with lentiviral particles (3ng p24/1000 cells) and underwent spinoculation (1000 × *g* for 10min). MDMs were also co-transduced with lentivectors expressing Vpx, produced as described before using VSV-G, HIV-1 gag-pol and the packaging construct pSIV3^+^.

### Production of WNV reporter virus particles (RVPs)

The constructs (WNVII Rep-G/Z and WNV NY99 CprME) used to generate the eGFP-encoding WNV RVPs were kindly provided by Theodore Pierson (Vaccine Research Center, National Institute of Allergy and Infectious Diseases, National Institutes of Health, Bethesda, Maryland, USA). Production of RVPs was performed as described previously [52]. Briefly, HEK293T cells were transfected with the plasmids encoding the replicons and the WNV structural proteins using PEI in a 3:1 (structural:replicon) ratio. RVP-containing supernatants were harvested 48 h post-transfection, filtered, concentrated by ultracentrifugation at 60,000 × *g* on an Optima XE-90 centrifuge with a SW 32 Ti rotor (Beckman Coulter), and stored at −80°C. Viral titers were determined by flow cytometry.

### *In vitro* transcription of NanoLuc replicons

Plasmids encoding flaviviral replicons in which the structural genes were replaced with NanoLuc (namely pCCI-DENV2-rep-Nanoluc, pCCI-ZIKV-rep-Nanoluc, pCCI-KUNV-rep-Nanoluc, pCCI-TBEV-rep-Nanoluc) have been previously described in [70]. An SP6 promoter sequence was added upstream of the NanoLuc gene by PCR using Phusion High Fidelity DNA Polymerase (Thermo Scientific) with appropriate primers (Table 4) on plasmid pLVX-NanoLuc, a kind gift of P.-O. Vidalain (CIRI, Lyon, France) previously described in [71]. All replicons and Nanoluc-expressing DNA regions were amplified by PCR using appropriate primers (Table 4) with Phusion High-Fidelity DNA Polymerase (Thermo Scientific) on a Mastercycler Gradient (Eppendorf) with the following program: 98°C for 2 min, then 35 cycles of denaturation at 98°C for 10s, hybridization at 60°C (for ZIKV replicon and Nanoluc) or 72°C (for DENV2, KUNV and TBEV replicons) for 15 s, elongation at 72°C for 7 min, and a final elongation at 72°C for 10 min. PCR products were purified using Wizard SV Gel and PCR Clean-Up System kit (Promega) according to manufacturer’s instructions. Purified linear DNA was *in vitro* transcribed and capped using mMESSAGE mMACHINE SP6 Transcription Kit (Invitrogen) according to manufacturer’s instructions. Transcribed RNA was purified using Rneasy kit (Qiagen) with an on-column Dnase digestion using RNase-Free DNase Set (Qiagen) according to manufacturer’s instructions, and stored at -80°C. At each step, RNA and DNA concentration and purity were evaluated by spectrophotometry (NanoDrop 2000c, Thermo Fisher Scientific) and migration in a 0.8% agarose gel for 30 min at 100V on a Mupid One Electrophoresis System Complete Apparatus (Eurogentec).

**Table 4:** Sequences of primers used for PCR prior to *in vitro* transcription.

Template plasmid	Direction	Primer sequence (5’ → 3’)
pCCI-DENV2-rep-Nanoluc	F	ACGCGTATTTAGGTGACACTATAGAGTTGTTAG
	R	AGAACCTGTTGATTCAACAGCACCATTCC
pCCI-ZIKV-rep-Nanoluc	F	GAATTCATTTAGGTGACACTATAGAGTTGTTG
	R	AGACCCATGGATTTCCCCACACCG
pCCI-KUNV-rep-Nanoluc	F	ACGCGTATTTAGGTGACACTATAGAGTAGTTCG
	R	AGATCCTGTGTTCTCGCACCACCAGC
pCCI-TBEV-rep-Nanoluc	F	AAACATTTAGGTGACACTATAGAGATTTTCTTGCACGTG
	R	AGCGGGTGTTTTTCCGAGTCACACATCAC
pLVX-Nanoluc	F	AAGATTTAGGTGACACTATAGGCCACCATGGTCTTCACACTCGAAGATTTCG
	R	TTACGCCAGAATGCGTTCGCACAG

### Quantification of flaviviral translation

HEK293T cells were transfected with *in vitro* transcribed NanoLuc-expressing RNA using Lipofectamine 2000 (Invitrogen). Eight hours post-transfection, cells were washed with PBS. NanoLuc activity was measured using Nano-Glo Luciferase Assay System (Promega) according to manufacturer’s instruction and detected on an Infinite M Plex (Tecan) instrument operated by Tecan i-control software (Tecan).

### Titration of secreted interferon

Cell supernatants were collected at the indicated time post-treatment. IFN was quantified on STING-37 cells that express the luciferase reporter under an ISRE (interferon-sensitive response element) promoter [65]. Briefly, STING-37 cells were exposed to the supernatants for 24 h in triplicate. A 10-fold serial dilution of Universal IFN-I (PBL Assay Science) was prepared to generate a standard curve. Luciferase substrate (Bright-Glo, Promega) was added directly into the wells and luciferase activity was measured on an Infinite M Plex (Tecan) instrument operated by Tecan i-control software (Tecan).

### Quantification and statistical analyses

Graphical representations and statistical analyses were performed using GraphPad Prism version 10 for Windows (GraphPad Software). To analyze two conditions, two-tailed Student’s tests were performed. To analyze multiple conditions, a normality test was first performed, and data were then analyzed with one-way or two-way analyses of variance (ANOVA) followed by multiple comparison analyses, as indicated in Fig legends.

## Supporting information

S1 Table Raw data and corresponding calculations supporting Fig 3, panels A-C. (XLSX)

S1 File **S1 Fig. SAMD9L restricts replication of multiple DENV serotypes.** Vero E6 cells were transduced with lentiviruses expressing SHFL or SAMD9L for 72 h, then infected with DENV-3 or DENV-4 (MOI = 1) for an additional 72 h. Infection and transduction efficiencies were quantified by flow cytometry, and infection levels were normalized to the empty vector control. Each dot represents a biological replicate. Data are shown as mean ± SEM. Statistical analysis was performed using one-way ANOVA with Dunnett’s multiple comparisons test. Statistical significance: ****, p ≤ 0.0001; ***, p ≤ 0.001. **S2 Fig. Validation of SAMD9 and SAMD9L silencing in HMC3 cells.** HMC3 microglial cells were transduced with lentiviral vectors expressing the indicated shRNAs for 48 h. Knockdown efficiency of SAMD9 and SAMD9L was assessed by RT-qPCR (**A**) and Western blot (**B**). Each dot represents a biological replicate. Data are shown as mean ± SEM. Statistical analysis was performed using two-way ANOVA with Tukey’s multiple comparisons test. Statistical significance: ****, p ≤ 0.0001; **, p ≤ 0.01. **S3 Fig. Phenotyping of human primary monocyte-derived macrophages (MDMs).** (**A**) Gating strategy. (**B**) Expression of surface markers on MDMs. Unstained control is in black. **S4 Fig. Validation of SAMD9 and SAMD9L silencing in monocyte-derived macrophages (MDMs).** Primary MDMs were transduced with lentiviral vectors expressing the indicated shRNAs, and knockdown efficiency of SAMD9 and SAMD9L was assessed 48 h post-transduction by RT-qPCR. Each dot represents a biological replicate. Data are shown as mean ± SEM. Statistical analysis was performed using two-way ANOVA with Tukey’s multiple comparisons test. Statistical significance: ****, p ≤ 0.0001; **, p ≤ 0.01; *, p ≤ 0.05. **S5 Fig. Expression of SAMD9 or SAMD9L does not affect transfection efficiency or transcription.** (**A**) Schematic organization of GFP-encoding replicons and plasmid. (**B**) HEK293T cells were co-transfected with the indicated expression plasmids for 48 h as described in Fig 3B, and GFP mRNA expression was assessed by RT-qPCR. (**C**) mCherry expression was analyzed by flow cytometry 48 h post-transfection in the same experiment as in Fig 3B. For each expression plasmid, data from co-transfections with the different GFP-encoding plasmids or replicons were pooled. Each dot represents a biological replicate. Data are shown as mean ± SEM. **S6 Fig. Validation of DNA/RNA quality and expression controls.** (**A**) HEK293T cells were transfected with the indicated N-terminally FLAG-mCherry-tagged (FmC) plasmids for 48 h, and mCherry expression was evaluated by flow cytometry prior to RNA transfection to confirm successful protein overexpression. (**B**) Schematic representations of the Nanoluc-encoding replicons used in the experiment. (**C**) Aliquots were collected at key steps of the workflow: after PCR amplification (DNA), after PCR product purification (p. DNA), after in vitro transcription (RNA), and after RNA purification (p. RNA). Samples were analyzed by agarose gel electrophoresis to assess nucleic acid integrity and purity. **S7 Fig. Wild-type SAMD9L overexpression induces an innate antiviral response.** (**A**) A549 cells were transfected with plasmids expressing N-terminally FLAG-mCherry-tagged (FmC) SAMD9 or SAMD9L (wt or LoF) for 24 h. Cells were treated or not with 10 µg/mL of CHX 8 h before cell lysis. Expression of CXCL10, SAMD9 and SAMD9L was quantified by RT-qPCR. (**B**) A549 cells were transfected with plasmids expressing untagged SAMD9 or SAMD9L (wt or LoF) for 24 h. Cells were treated or not with 10 µg/mL of CHX 8 h before cell lysis. Expression of CXCL10, SAMD9 and SAMD9L was quantified by RT-qPCR. (**C**) A549 cells were transduced with lentiviral particles expressing untagged SAMD9L for 48 h, and transcript levels of IFNs, ISGs, and chemokines were quantified by RT-qPCR. Empty vector (SCRPSY backbone) was used as a control. (**D**) IFN-I levels in the supernatants of transduced cells were assessed using a luciferase-based assay. Each dot represents a biological replicate. Data are presented as mean ± SEM. Statistical analyses: one-way ANOVA with Dunnett’s multiple comparisons test (**A-B**), and unpaired t-test (**C–D**). Statistical significance: ****, p ≤ 0.0001; ***, p ≤ 0.001; **, p ≤ 0.01; *, p ≤ 0.05; ns, p > 0.5. Abbreviations: CHX, cycloheximide; IFN-I, type I interferon; E2C, E2 Crimson. **S8 Fig. Overexpression of SAMD9L does not elicit an innate antiviral response in HEK293T cells.** (**A**) HEK293T cells were transfected with plasmids expressing N-terminally FLAG-mCherry-tagged (FmC) SAMD9 or SAMD9L (wt or LoF) for 24 h. Cells were treated or not with 10 µg/mL of CHX 8 h before cell lysis. Expression of IFNs, ISGs, and chemokines was quantified by RT-qPCR. (**B**) IFN-I secretion was measured in supernatants from the same transfected cells using a luciferase-based assay 48 h post-transfection. (**C**) HEK293T cells were transfected with plasmids expressing untagged SAMD9 or SAMD9L (wt or LoF) for 24 h. Cells were treated or not with 10 µg/mL of CHX 8 h before cell lysis. Expression of IFNs, ISGs, and chemokines was quantified by RT-qPCR. (**D**) IFN-I levels in the supernatants of transfected cells were measured using a luciferase-based assay 48 h post-transfection. Each dot represents a biological replicate. Data are presented as mean ± SEM. Statistical analyses: one-way ANOVA with Dunnett’s multiple comparisons test (**A-D**). Statistical significance: ****, p ≤ 0.0001; ***, p ≤ 0.001; **, p ≤ 0.01; *, p ≤ 0.05; ns, p > 0.5. Abbreviations: CHX, cycloheximide; E2C, E2 Crimson; IFN-I, type I interferon. **S9 Fig. Untagged human SAMD9L inhibits translation of a GFP-encoding WNV-based replicon.** HEK293T cells were co-transfected with either E2 Crimson (control) or untagged wt SAMD9, SAMD9 LoF, wt SAMD9L, or SAMD9L LoF expression plasmids, along with a GFP-expressing WNV replicon (WNVII Rep-G/Z). GFP and SAMD9/SAMD9L expression were analyzed by Western blot 48 h post-transfection. **S10 Fig. Validation of ISG expression inhibition by Ruxolitinib.** A549 cells were treated with 10 µM Ruxolitinib or DMSO (vehicle control). Six hours later, cells were stimulated with 100 U/mL of type I interferon (IFN-I) for 24 h. Expression levels of interferon-stimulated genes (ISGs) were measured by RT-qPCR. Each dot represents a biological replicate. Data are shown as mean ± SEM. Statistical analysis was performed using one-way ANOVA followed by Tukey’s multiple comparisons test. Statistical significance: ****, p ≤ 0.0001; ***, p ≤ 0.001; **, p ≤ 0.01. Abbreviations: IFN-I, type I interferon; Ruxo, Ruxolitinib.
(PDF)
