# Supplementary material for: SAMD9L inhibits flavivirus translation independently of its capacity to trigger innate immune response
Source: PLoS Pathog. 2025 Dec 8;21(12):e1013773. doi: 10.1371/journal.ppat.1013773 (PMC12698002; doi:10.1371/journal.ppat.1013773)

Supporting information

Fig S1. SAMD9L restricts replication of multiple DENV serotypes.

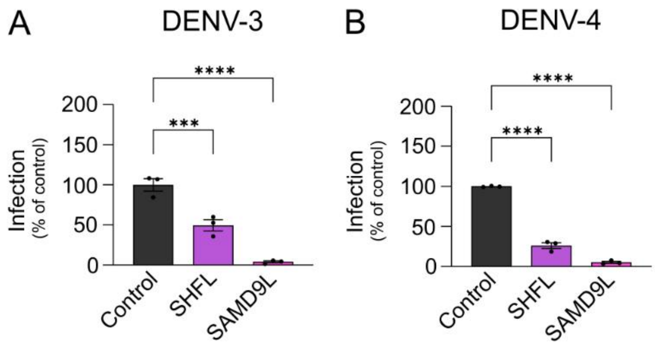

Fig S2. Validation of SAMD9 and SAMD9L silencing in HMC3 cells.

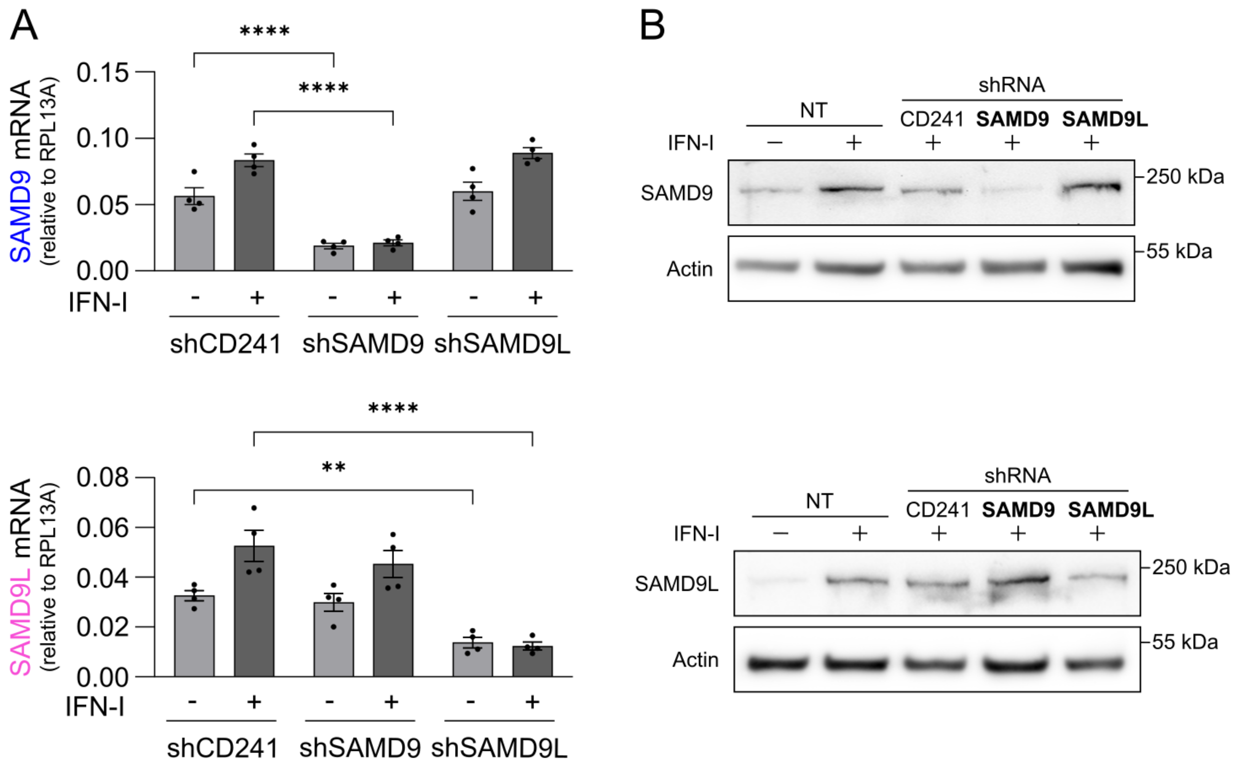

Fig S3. Phenotyping of human primary monocyte-derived macrophages (MDMs).

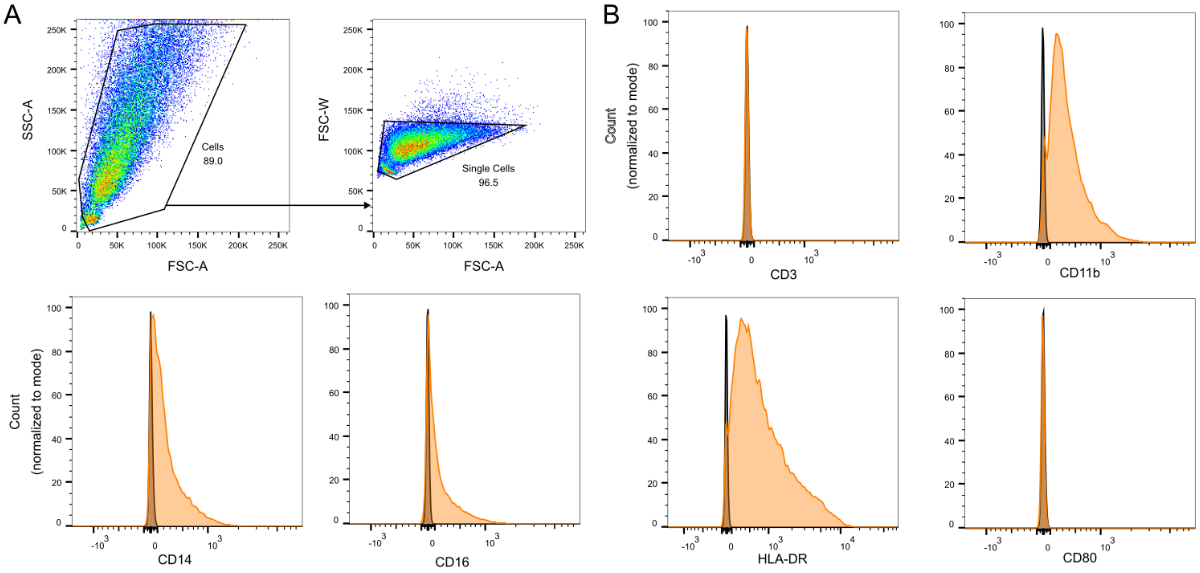

Fig S4. Validation of SAMD9 and SAMD9L silencing in monocyte-derived macrophages (MDMs).

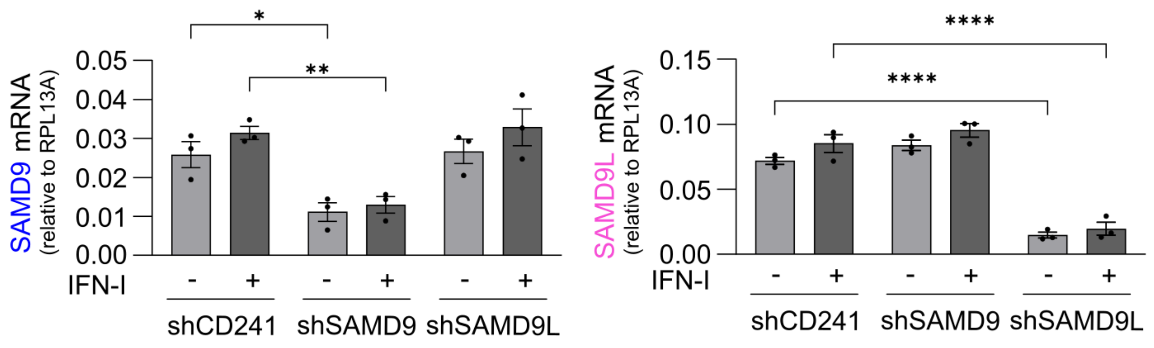

Fig S5. Expression of SAMD9 or SAMD9L does not affect transfection efficiency or transcription.

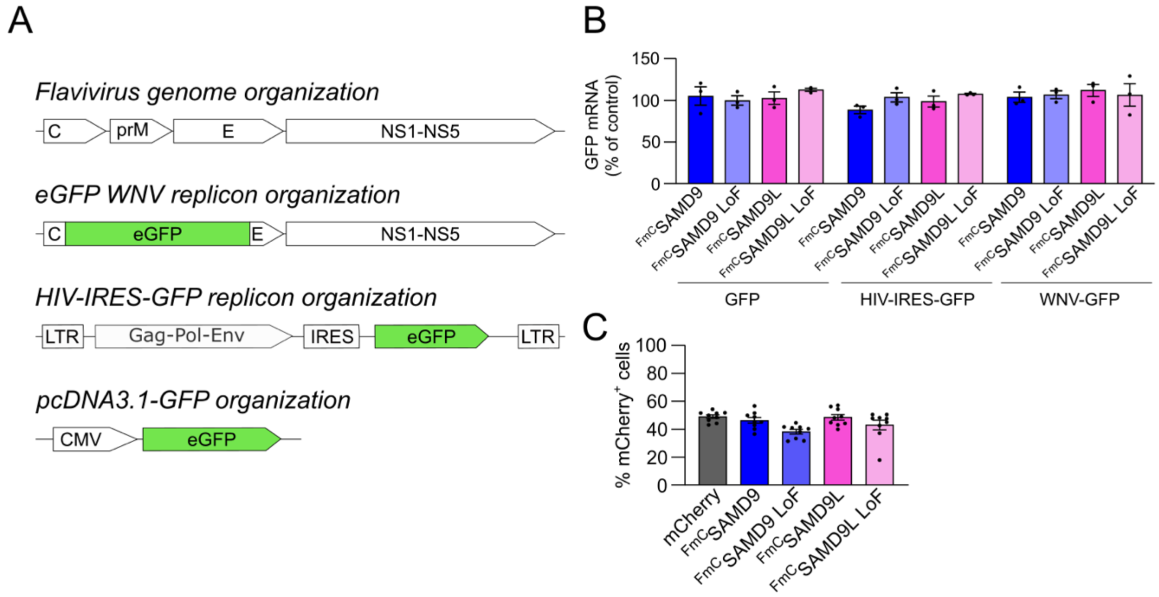

Fig S6. Validation of DNA/RNA quality and expression controls.

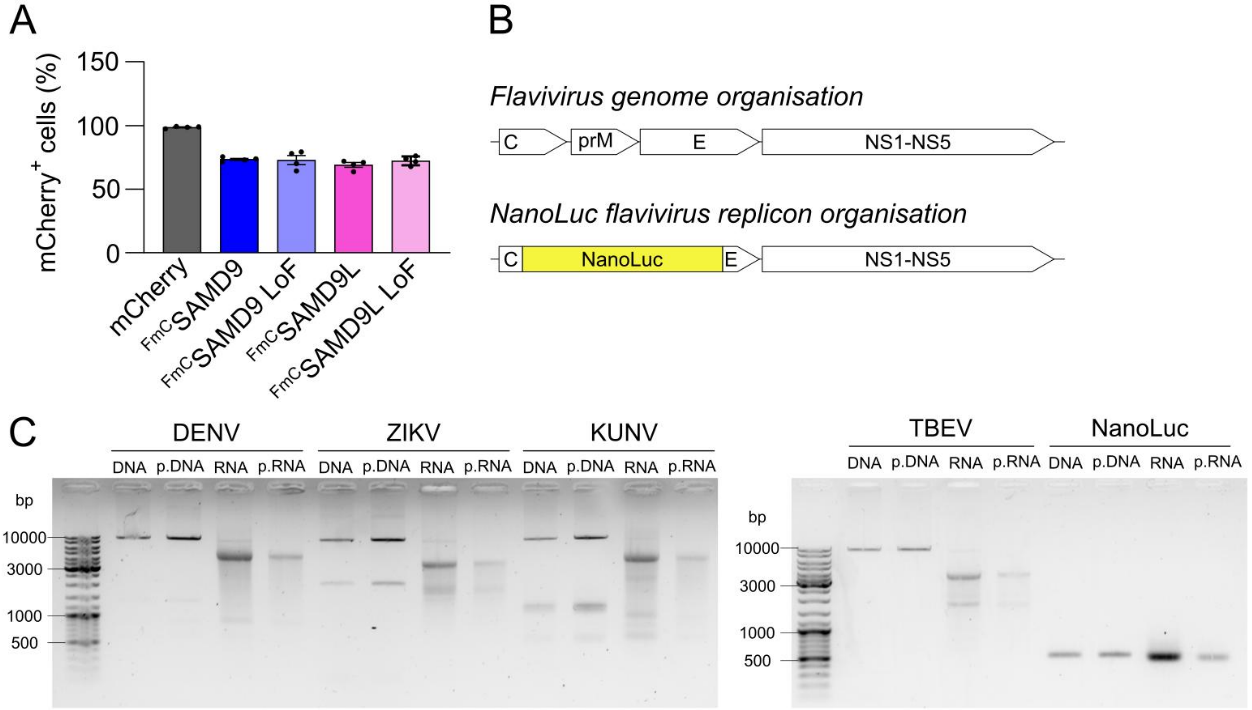

Fig S7. Wild-type SAMD9L overexpression induces an innate antiviral response.

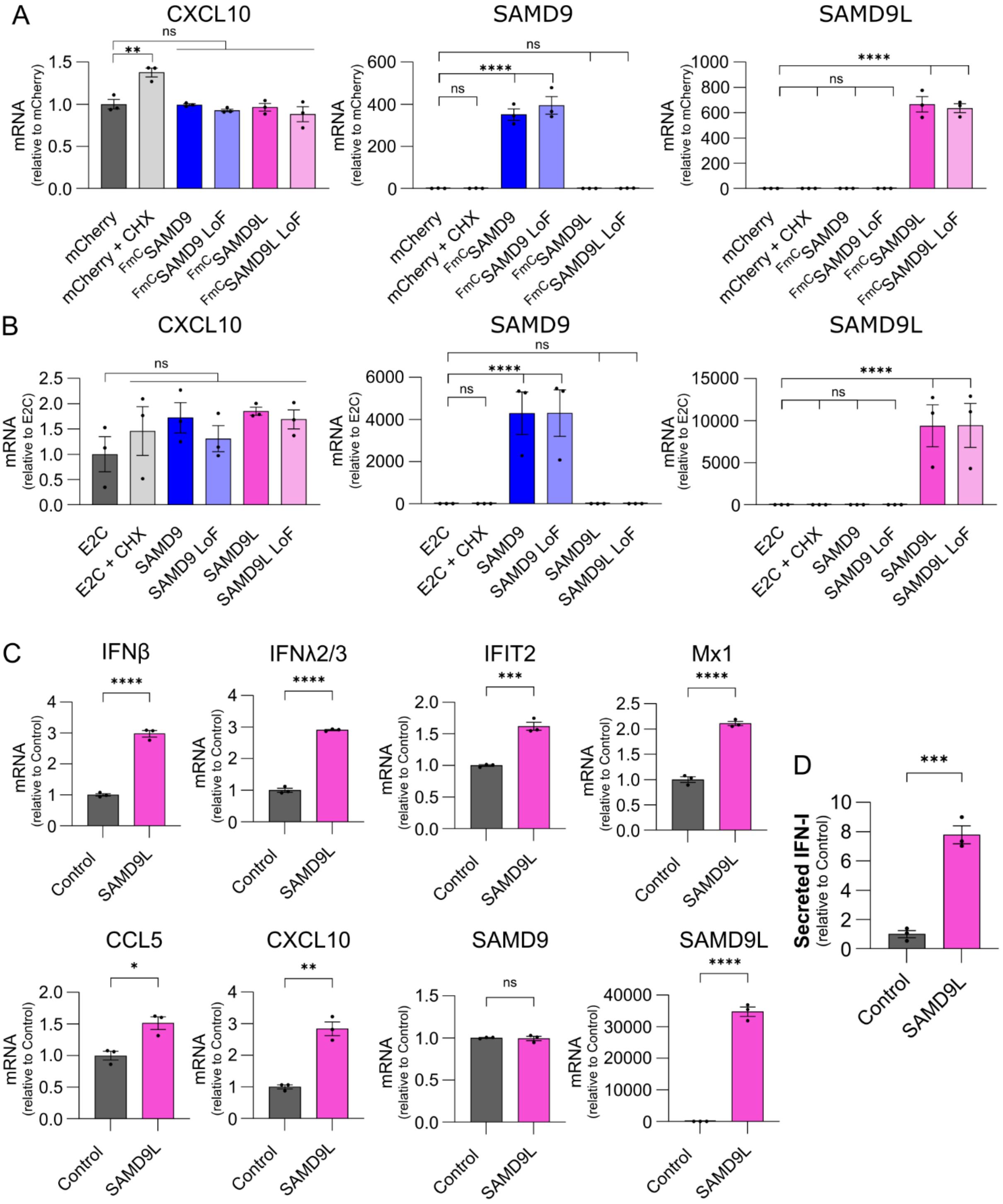

Fig S8. Overexpression of SAMD9L does not elicit an innate antiviral response in HEK293T cells.

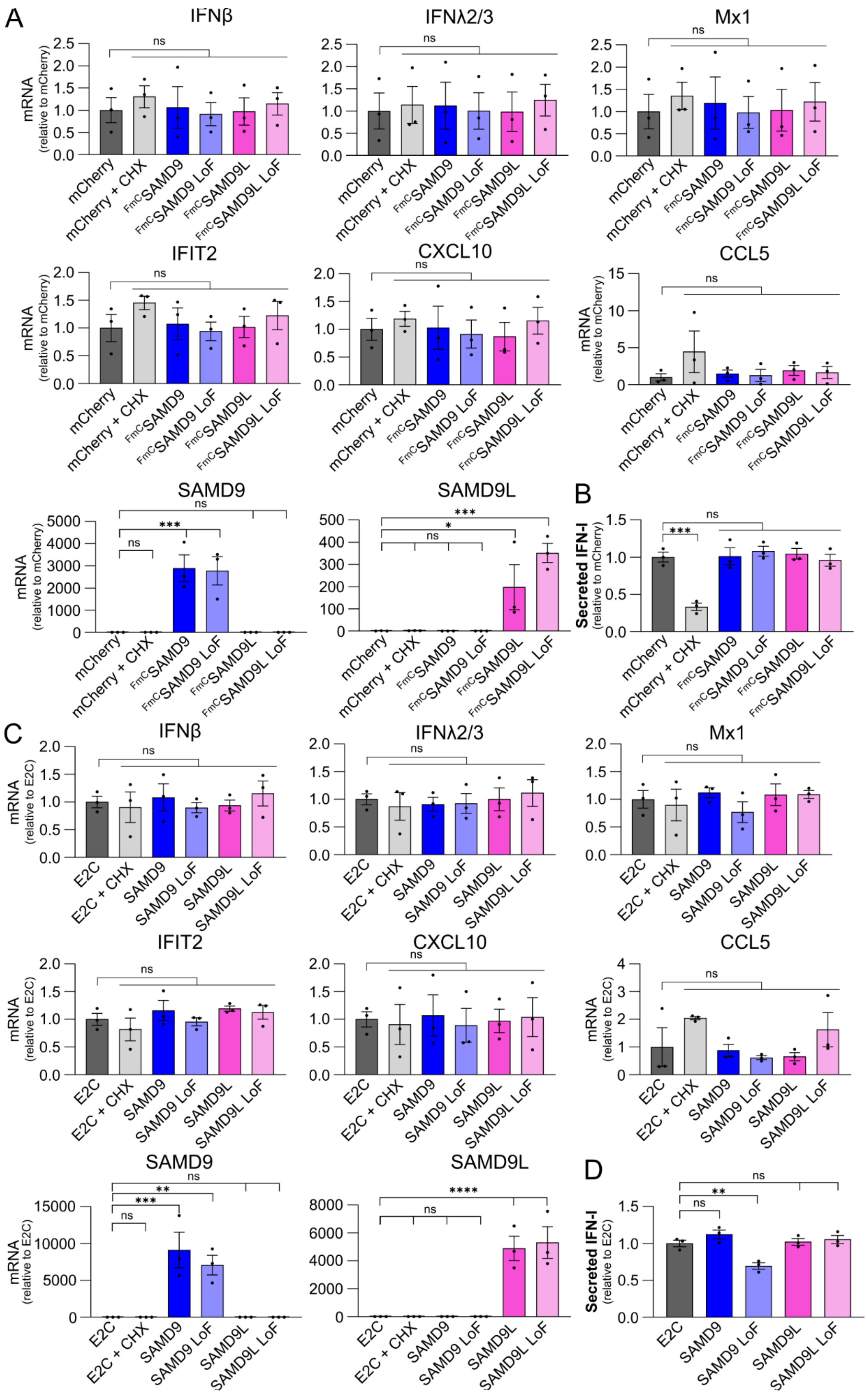

Fig S9. Untagged human SAMD9L inhibits translation of a GFP-encoding WNV-based replicon.

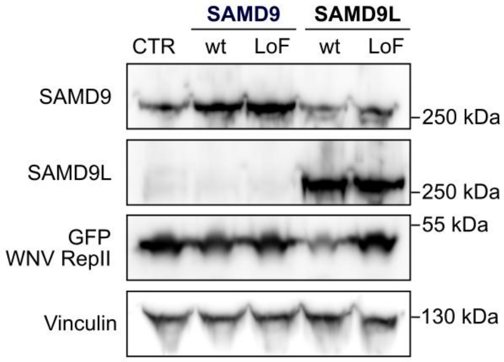

Fig S10. Validation of ISG expression inhibition by Ruxolitinib.

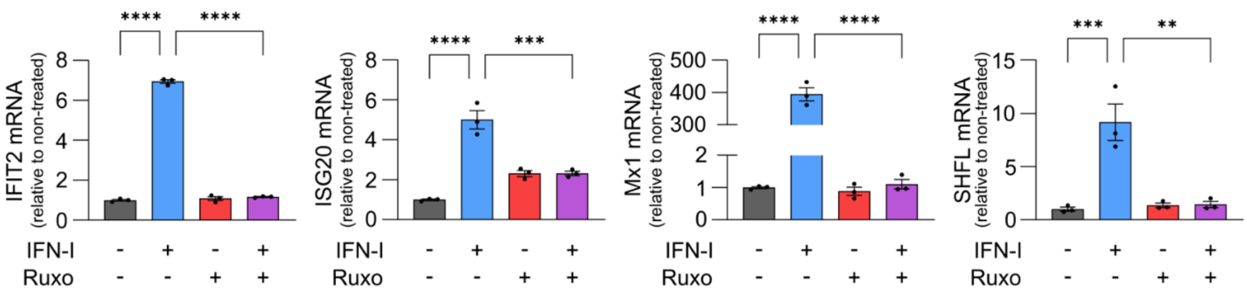

Supplement: S1 File — S1 Fig. SAMD9L restricts replication of multiple DENV serotypes. Vero E6 cells were transduced with lentiviruses expressing SHFL or SAMD9L for 72 h, then infected with DENV-3 or DENV-4 (MOI = 1) for an additional 72 h. Infection and transduction efficiencies were quantified by flow cytometry, and infection levels were normalized to the empty vector control. Each dot represents a biological replicate. Data are shown as mean ± SEM. Statistical analysis was performed using one-way ANOVA with Dunnett’s multiple comparisons test. Statistical significance: ****, p ≤ 0.0001; ***, p ≤ 0.001. S2 Fig. Validation of SAMD9 and SAMD9L silencing in HMC3 cells. HMC3 microglial cells were transduced with lentiviral vectors expressing the indicated shRNAs for 48 h. Knockdown efficiency of SAMD9 and SAMD9L was assessed by RT-qPCR (A) and Western blot (B). Each dot represents a biological replicate. Data are shown as mean ± SEM. Statistical analysis was performed using two-way ANOVA with Tukey’s multiple comparisons test. Statistical significance: ****, p ≤ 0.0001; **, p ≤ 0.01. S3 Fig. Phenotyping of human primary monocyte-derived macrophages (MDMs). (A) Gating strategy. (B) Expression of surface markers on MDMs. Unstained control is in black. S4 Fig. Validation of SAMD9 and SAMD9L silencing in monocyte-derived macrophages (MDMs). Primary MDMs were transduced with lentiviral vectors expressing the indicated shRNAs, and knockdown efficiency of SAMD9 and SAMD9L was assessed 48 h post-transduction by RT-qPCR. Each dot represents a biological replicate. Data are shown as mean ± SEM. Statistical analysis was performed using two-way ANOVA with Tukey’s multiple comparisons test. Statistical significance: ****, p ≤ 0.0001; **, p ≤ 0.01; *, p ≤ 0.05. S5 Fig. Expression of SAMD9 or SAMD9L does not affect transfection efficiency or transcription. (A) Schematic organization of GFP-encoding replicons and plasmid. (B) HEK293T cells were co-transfected with the indicated expression plasmids for 48 [file ppat.1013773.s002.pdf]
